# A Liposomal Drug Platform Overrides Peptide Ligand Targeting to a Cancer Biomarker, Irrespective of Ligand Affinity or Density

**DOI:** 10.1371/journal.pone.0072938

**Published:** 2013-08-23

**Authors:** Bethany Powell Gray, Michael J. McGuire, Kathlynn C. Brown

**Affiliations:** Department of Internal Medicine and The Simmons Comprehensive Cancer Center, University of Texas Southwestern Medical Center, Dallas, Texas, United States of America; Texas Tech University Health Sciences Center, United States of America

## Abstract

One method for improving cancer treatment is the use of nanoparticle drugs functionalized with targeting ligands that recognize receptors expressed selectively by tumor cells. In theory such targeting ligands should specifically deliver the nanoparticle drug to the tumor, increasing drug concentration in the tumor and delivering the drug to its site of action within the tumor tissue. However, the leaky vasculature of tumors combined with a poor lymphatic system allows the passive accumulation, and subsequent retention, of nanosized materials in tumors. Furthermore, a large nanoparticle size may impede tumor penetration. As such, the role of active targeting in nanoparticle delivery is controversial, and it is difficult to predict how a targeted nanoparticle drug will behave *in vivo*. Here we report *in vivo* studies for α_v_β_6_-specific H2009.1 peptide targeted liposomal doxorubicin, which increased liposomal delivery and toxicity to lung cancer cells *in vitro*. We systematically varied ligand affinity, ligand density, ligand stability, liposome dosage, and tumor models to assess the role of active targeting of liposomes to α_v_β_6_. In direct contrast to the *in vitro* results, we demonstrate no difference in *in vivo* targeting or efficacy for H2009.1 tetrameric peptide liposomal doxorubicin, compared to control peptide and no peptide liposomes. Examining liposome accumulation and distribution within the tumor demonstrates that the liposome, and not the H2009.1 peptide, drives tumor accumulation, and that both targeted H2009.1 and untargeted liposomes remain in perivascular regions, with little tumor penetration. Thus H2009.1 targeted liposomes fail to improve drug efficacy because the liposome drug platform prevents the H2009.1 peptide from both actively targeting the tumor and binding to tumor cells throughout the tumor tissue. Therefore, using a high affinity and high specificity ligand targeting an over-expressed tumor biomarker does not guarantee enhanced efficacy of a liposomal drug. These results highlight the complexity of *in vivo* targeting.

## Introduction

Cancer is the number one cause of death in the world, and the number of cancer related deaths is projected to rise in the coming decades [1]. One paradigm for improving cancer treatment is the development of targeting therapies that use tumor-specific ligands to selectively deliver drugs to cancer cells, thus increasing drug accumulation in the tumor and decreasing unwanted toxicities from drug accumulation in other areas of the body. Tumor-specific ligands accumulate preferentially in tumors due to specificity for a receptor expressed selectively by the tumor or tumor vasculature cells (and not expressed by normal cells). Nanoparticle drugs are particularly attractive for use with tumor-specific ligands due to encapsulation of the drug within a nanoparticle, which prevents drug activity until its release from the nanoparticle and can increase blood circulation time.

Pegylated liposomal doxorubicin (DOXIL®/CAELYX®) was the first nanoparticle clinically approved for cancer treatment. DOXIL® is approximately 100 nm in size, contains the anthracycline chemotherapeutic doxorubicin [2], and is currently approved for the treatment of ovarian cancer [3], multiple myeloma [4], and Kaposi’s sarcoma [5] in both the United States and Europe and for use in breast cancer patients in Europe [6]. Numerous clinical trials involving the drug are ongoing, including trials in patients with non-small cell lung cancer (NSCLC) [7]. Due to the clinical success of DOXIL®, most targeting ligands conjugated to nanoparticles for targeted drug delivery have been conjugated to liposomal forms of doxorubicin. Both antibody and peptide targeting ligands have been used to increase the efficacy and decrease the toxicity of liposomal doxorubicin by actively targeting tumor and tumor vasculature cells [8–25]. Of particular interest, anti-HER2 liposomal doxorubicin and anti-EGFR liposomal doxorubicin formulations are in Phase I clinical trials [26,27]. Additionally, liposomal doxorubicin conjugated to a peptide derivative of the tumor vasculature targeting NGR peptide [28] has been primed for potential future clinical trials by preparation using Good Manufacturing Practices (GMP) [29].

Included among the many advantages of using liposomal doxorubicin for targeting therapies is the high drug to targeting ligand ratio due to the thousands of doxorubicin molecules trapped inside each liposome. Additionally, pegylated liposomal doxorubicin enjoys long *in vivo* circulation times [30], extending the time targeting ligands have to deliver their cargo to the tumor. An important factor contributing to liposomal drug efficacy is the passive accumulation of nano-sized particles in the tumor through the enhanced permeability and retention (EPR) effect [31]. Unlike the vasculature of normal tissue, tumor vasculature is irregular and disordered. Nano-sized particles can escape through this leaky vasculature into the surrounding tumor tissue and are subsequently retained within the tumor due to the poor lymphatic drainage systems of tumors. Thus tumor accumulation of ligand-targeted liposomes depends not only on the specific targeting ligand but also on EPR-driven effects. The role of active targeting in delivery of nanoparticles to tumors is controversial [32–36].

Studies with targeted liposomes have demonstrated two mechanisms for improved tumor drug accumulation, both of which lead to desirable therapeutic outcomes. Some peptide-targeted liposomes, in particular those targeting the tumor vasculature, including the NGR-liposomes, deliver more doxorubicin to the tumors than non-targeted liposomes [9,16], suggesting that the targeted liposomes are accumulating in the tumor based on both the peptide targeting abilities and the EPR effect. Other antibody-targeted liposome formulations, including anti-HER2 and anti-EGFR liposomes, accumulate in the tumor at levels similar to non-targeted liposomes. However unlike the non-targeted liposomes, these targeted liposomes internalize into tumor cells and exhibit better distribution throughout the tumor tissue [37,38]. While the targeting ligand does not override the EPR effect driving tumor accumulation of these liposomal formulations, the altered location of the drug to its site of action inside the tumor cells increases efficacy. Due to the EPR effect and the different vasculature structure of every tumor, it is difficult to predict how an untested targeting liposome formulation will accumulate in tumors and affect tumor growth.

We recently described the development of peptide targeted liposomal doxorubicin formulations specific for the restrictively expressed receptor α_v_β_6_ [39]. The integrin α_v_β_6_ is emerging as an ideal cancer target; it is expressed by a variety of cancers of the epithelium [40–50] and only rarely expressed in normal tissue [51]. Of particular interest is the prevalence of α_v_β_6_ in lung cancer, the number one cancer killer of both men and women [1]. More than half of patient non-small cell lung cancer (NSCLC) tumors express α_v_β_6_, and integrin expression is “turned on” during the early stages of NSCLC carcinogenesis and remains elevated throughout the progression of the disease [52].

We have identified and subsequently optimized an α_v_β_6_-specific peptide, H2009.1 [53]. The monomeric peptide binds H2009 adenocarcinoma NSCLC cells with a half-maximal binding affinity of 9.2 nM, and synthesizing the H2009.1 peptide as a phage structure-mimicking tetrameric peptide increases affinity to 11pM [54]. Both the monomeric and tetrameric peptides internalize into α_v_β_6_-expressing cells *in vitro* and target tumors *in vivo*. With the goal of translating peptides isolated from phage display libraries into effective therapeutic delivery agents, we examined the best platform for displaying the H2009.1 peptides on liposomal doxorubicin for drug delivery *in vitro*, varying both liposomal peptide density and peptide valency [39]. *In vitro*, the most effective liposome formulation displays the H2009.1 tetrameric peptide at a density of 1.3% of the total lipid content. The 1.3% H2009.1 tetrameric liposome formulation was 2-fold more toxic to cells than liposomes bearing the control scrambled scH2009.1 peptide and more than 10 times more toxic than naked, no peptide, liposomes. Despite these *in vitro* results, it is unclear whether the same liposome formulation will best target α_v_β_6_-expressing tumors *in vivo*. Significant differences exist between the *in vitro* to *in vivo* contexts, including possible differences in receptor expression and availability, tumor vasculature structure, and drug biodistribution effects.

To examine the best H2009.1 targeted liposomal doxorubicin formulation for inhibiting α_v_β_6_-expressing tumor growth *in vivo*, we systematically varied ligand affinity, ligand density, ligand stability, liposome dosage, and tumor models to assess the role of active targeting of the liposome in three NSCLC models. Despite the targeting differences between the different liposome formulations *in vitro*, all of the H2009.1 peptide liposome platforms exhibit identical efficacy *in vivo*. Additionally, there is no efficacy difference between the H2009.1 liposomes and control no-peptide liposomes. Subsequent experiments demonstrate that this result is due to EPR-based liposome tumor accumulation and failure of the liposomes to penetrate the tumor tissue past areas immediately adjacent to the tumor vasculature, despite widespread expression of α_v_β_6_ in the tumor. These results highlight the complexity of *in vivo* drug targeting, even when using a high affinity ligand known to selectively target tumors *in vivo*, and suggest that a large nanoparticle may not be the best targeting platform for every tumor environment.

## Materials and Methods

### Materials

All Fmoc amino acids were purchased from Novabiochem® (EMD Millipore, Billerica, MA). Sepharose CL-4B and Sephadex G-50 were purchased from Sigma-Aldrich Inc. (Livermore, CA). Lipids were purchased from Avanti® Polar Lipids, Inc. (Alabaster, AL) and doxorubicin HCl for injection from Bedford Laboratories™ (Bedford, OH). The Molecular Probes® dyes DiI [DiI(C)_18_(3), 1,1'-dioctadecyl-3,3,3',3'-tetramethylindocarbocyanine perchlorate] and DiR [DiOC_18_(7), 1,1'-dioctadecyl-3,3,3',3'-tetramethylindotricarbocyanine iodide], were purchased from Life Technologies™ (Grand Island, NY). For cell culture, fetal bovine serum (FBS) was purchased from Gemini Bio-Products (West Sacramento, CA) and both RPMI 1640 and Trypsin EDTA, 1x from Mediatech, Inc. (Fisher Scientific, Pittsburgh, PA).

### Cell Lines

All human NSCLC cell lines used were previously established and characterized [55]. Cell lines were obtained from the Hamon Center for Therapeutic Oncology Research at UT Southwestern Medical Center. Cell lines were routinely tested for Mycoplasma and DNA fingerprinted to confirm their identity. The H2009, H1975, and H460 cell lines were all grown at 37°C and 5% CO_2_ in RPMI 1640 supplemented with 5% FBS.

### Peptide Synthesis and Purification

Both monomeric and tetrameric H2009.1 and scH2009.1 peptides were synthesized as previously described [54]. Briefly, all monomeric peptides and the tetrameric core needed to make the tetrameric peptides were synthesized on a Symphony Synthesizer (Rainin Instruments, Protein Technologies, Inc., Tucson, AZ) by standard Fmoc solid-phase peptide synthesis. The tetrameric peptides were synthesized by reaction of 5-fold excess of purified cysteine-bearing monomeric peptides with purified maleimide activated tetrameric core, in a solution of PBS + 10mM EDTA with shaking at RT for 2 hours. All peptides were purified by reverse phase high-performance liquid chromatography (HPLC) using a SPIRIT™ Peptide C18 5 µm, 25 x 2.12 column (AAPPTec®, Louisville, KY) on a Breeze™ HPLC (Waters Corporation, Milford, MA) according to published elution conditions [54]. Matrix-assisted laser desorption/ionization time of flight mass spectrometry was used to confirm peptide mass (Voyager-DE™ PRO, Applied Biosystems, Inc., Foster City, CA). The masses of the monomeric peptides (average mass calculated/MH+: 1843.02/1844.18) and tetrameric core (average mass calculated/MNa+: 1251.49/1274.27) were determined in reflective mode using α-cyano-4-hydroxycinnamic acid as a matrix. The masses of the tetrameric peptides (average mass calculated/MH+: 8629.01/8626.77) were determined in linear mode using sinapinic acid as a matrix.

### Preparation of Liposomal Doxorubicin

Liposomes were prepared from solutions of the lipids hydrogenated soy L-α-phophatidylcholine (HSPC), cholesterol, 1,2-distearoyl-sn-glycero-3-phosphoethanolamine-N-[carbonyl-methoxy(polyethylene glycol)-2000] (DSPE-PEG_20000_), and 1,2-distearoyl-sn-glycero-3-phosphoethanolamine-N-[maleimide(polyethylene glycol)-2000] (DSPE-PEG_2000_-maleimide) in 2:1 chloroform: methanol. The 0.64% liposomes were prepared from mixtures of 100 mg (131 µmol) of HSPC, 25.4 mg (65.6 µmol) of cholesterol, 14.7 mg (5.20 µmol) of DSPE-PEG_20000_ and 3.85 mg (1.31 µmol) of DSPE-PEG_2000_-maleimide. The 1.3% liposomes were prepared from mixtures of 100 mg (131 µmol) of HSPC, 25.4 mg (65.6 µmol) of cholesterol, 10.9 mg (3.87 µmol) of DSPE-PEG_20000_ and 7.92 mg (2.69 µmol) of DSPE-PEG_2000_-maelimide. Solvent was removed under a slow stream of nitrogen at 45^°^C, and the lipid film was left under vacuum overnight. The dried lipid film was hydrated with 155 mM (NH_4_)_2_SO_4_ buffer, pH 5.5, by intermittently heating at 65^°^C and vortexing. Liposomes were subsequently extruded 20 times through double-stacked 100nm membranes, and PD-10 desalting columns (GE Healthcare, Waukesha, WI) were used to change the outer liposomal buffer to 123 mM NaCitrate, pH 5.5. Doxorubicin was loaded remotely (post-liposome formation) by incubation of liposomes and doxorubicin at 65^°^C for 1 hour. Free doxorubicin was removed using a Sephadex G-50 column equilibrated with HEPES buffered saline. Peptides were conjugated to liposomes by reaction for 24 hours at a ratio of 2:1 peptide: DSPE-PEG_2000_-maelimide in a solution of HEPES buffered saline, and excess peptide was removed using Sepharose CL-4B columns.

### Preparation of DiI or DiR-Labeled Liposomes

Dye labeled liposomes were prepared as described except that they were not loaded with doxorubicin. The 1.3% liposome formulation was prepared with the addition of the DiI or DiR dye into the mixture of lipids in 2:1 chloroform: methanol. The DiI or DiR dyes were dissolved in ethanol at a concentration of 2.5 mg/mL and were added to the lipid mixture at a ratio of 3.75 µg dye/0.5 mg lipid. Solvent was removed under a slow stream of nitrogen at 45^°^C, and the lipid film was left under vacuum overnight. The dried lipid film was hydrated with 155 mM (NH_4_)_2_SO_4_ buffer, pH 5.5, by intermittently heating at 65^°^C and vortexing. Liposomes were subsequently extruded 20 times through double-stacked 100 nm membranes, and PD-10 desalting columns (GE Healthcare, Waukesha, WI) were used to change the outer liposomal buffer to HEPES buffered saline. Peptides were conjugated to liposomes by reaction for 24 hours at a ratio of 2:1 peptide: DSPE-PEG_2000_-maelimide in a solution of HEPES buffered saline and excess peptide was removed using Sepharose CL-4B columns.

### Establishment of Mouse Tumor Models

Animal protocols were approved by the Institutional Animal Care and Use Committee at UT Southwestern Medical Center (Animal Welfare Assurance Number A3472-01, protocol number 2010-0280). All imaging was performed under isoflurane, and every effort was made to minimize suffering in accordance with the recommendations in the Guide for the Care and Use of Laboratory Animals of the National Institutes of Health. Female NOD/SCID mice (from the UT Southwestern Medical Center Mouse Breeding Core Facility) were injected with 1 million H2009, H1975, or H460 cells in the right flank. All cells were injected in phosphate buffered saline, pH 7.4, and were prepared for injection by incubating the cells with 0.05% Trypsin-EDTA (Gibco®, Life Technologies™, Grand Island, NY) for 10 minutes, quenching the trypsin with media, and washing the cells with phosphate buffered saline before final suspension in the phosphate buffered saline at a concentration of 10 million cells per mL.

### In Vivo Therapeutic Experiments

Subcutaneous H2009 tumors were established in the flank of NOD/SCID mice. Once palpable tumors had formed, 18 days after tumor cell implantation, the mice were treated with HBS (control) or different liposome formulations, based on the total concentration of doxorubicin. The different liposome formulations and treatment doses are described in detail in the Results section. For all experiments, mice were treated once weekly for 3 weeks, on days 18, 25, and 32, via tail vein injection. Tumors were measured by an independent scientist, and tumor volumes were calculated from the formula *V* = *(l x w* ^2^ *)*/2.

### Statistical Methods

The statistical significance of tumor size differences between drug treated groups and the control group were calculated using one way ANOVA with Dunnett’s multiple comparison test and between different drug treated groups, using one way ANOVA with Tukey’s multiple comparison test. The statistical significance of differences between survival curves were calculated from Kaplan-Meier curves with log-rank tests. All calculations were determined using GraphPad Prism.

### In Vivo and Ex Vivo Near Infrared Imaging

Mice bearing subcutaneous H2009, H1975, or H460 tumors in the right flank were injected via tail vein with DiR-labeled liposomes at a concentration of 22.22 µmol phospholipid/kg. This phospholipid/kg concentration correlates to the same amount of phospholipid (and therefore the same number of liposomes) as present in a treatment of 4 mg/kg liposomal doxorubicin. H2009 and H460 tumor-bearing mice were injected with DiR-labeled versions of the 1.3% H2009.1 tetrameric, AcH2009.1 tetrameric, scH2009.1 tetrameric, or naked liposomes with 3 mice per liposome group. Mice bearing H1975 tumors were injected with DiR-labeled versions of the 1.3% H2009.1 tetrameric, scH2009.1 tetrameric, or naked liposomes with 3 mice per liposome group. For each mouse, Nair® was used to remove all hair from the lower half of the body. At 24, 48, and 72 hours post-liposome injection, the mice were imaged for DiR dye fluorescence using an IVIS® Lumina (Caliper Life Sciences, Hopkinton, MA). After whole mouse imaging at 72 hours, all mice were sacrificed and organs removed for *ex vivo* fluorescent imaging. For each mouse, the organs were imaged on both sides, and the average of the radiant efficiency value of both sides used as the actual value of the liposome accumulation in that tissue.

### Microscopy of DiI Liposomes in Tumor Sections

Mice bearing subcutaneous H2009, H1975, or H460 tumors were injected via tail vein with DiI-labeled liposomes at a concentration of 22.22 µmol phospholipid/kg. For each tumor type, mice were injected with DiI-labeled versions of the 1.3% H2009.1 tetrameric, scH2009.1 tetrameric, or naked liposomes, with 3 mice per liposome group. One mouse from each treatment group was sacrificed at 24 hours, a second mouse at 48 hours, and the third mouse at 72 hours. Upon sacrifice, the tumors were removed and snap frozen inside cryomolds using Tissue-Tek® O.C.T. Compound mounting medium (Fisher Scientific, Pittsburgh, PA).

For microscopy, the tumors were sectioned at 10 µm using a Leica CM3050S cryostat (Leica Microsystems Inc., Buffalo Grove, IL). Sections were obtained from the top, middle, and bottom of each tumor. For the vasculature stain, a CD31 primary antibody (Rat AntiMouse CD31, catalog # 550274, BD Biosciences, San Jose, CA) was used at a 1:50 dilution and a fluorescein goat anti-rat secondary antibody (catalog # A10528, Life Technologies™, Grand Island, NY) was used at a 1:100 dilution. The slides were mounted with Dapi Fluoromount-G (SouthernBiotech, Birmingham, AL) and imaged on a Leica CTR5500 microscope (Leica Microsystems Inc., Buffalo Grove, IL) and a DeltaVision *pDV* deconvolution microscope (Applied Precision, Inc., Issaquah, WA).

### β_6_ Staining of Tumor Sections

Tumor sections prepared from the tumors containing DiI-liposomes were stained for β_6_ expression using a β_6_ primary antibody (catalog # MAB2076Z, EMD Millipore, Billerica, MA) at a 1:50 dilution and a fluorescein goat anti-rat secondary antibody (catalog # A10528, Life Technologies™, Grand Island, NY) at a 1:100 dilution. Slides were imaged on a Leica CTR5500 microscope (Leica Microsystems Inc., Buffalo Grove, IL) and a DeltaVision *pDV* deconvolution microscope (Applied Precision, Inc., Issaquah, WA).

## Results

### In Vivo Efficacy of H2009.1 Tetrameric Peptide Liposomes Targeting α_v_β_6_

To examine whether the α_v_β_6_-specific H2009.1 peptide can be used to increase the *in vivo* delivery and efficacy of liposomal doxorubicin towards α_v_β_6_-expressing NSCLC tumors, we set out to explore the effects of liposomal peptide density and valency on therapeutic results in tumor-bearing mice. We began by treating tumor-bearing mice with the 1.3% H2009.1 tetrameric peptide liposome formulation that demonstrated the best *in vitro* efficacy with limited non-specific toxicity [39]. In this 1.3% liposome formulation, 1.3% of the total lipid is modified with maleimide to allow cysteine-bearing peptides to couple to the lipid via cysteine-maleimide chemistry. Thus, 1.3% of the lipids comprising each liposome bear the H2009.1 peptide, resulting in approximately 1400 peptides per liposome. Coupling the H2009.1 tetrameric peptide to the 1.3% liposome formulation results in a nanoparticle-based multivalent display of the inherently multivalent tetrameric peptide, resulting in a layered multivalency that leads to higher specificity and greater toxicity towards α_v_β_6_-expressing NSCLC cells *in vitro* than liposomes modified with the monomeric H2009.1 peptide.

H2009 NSCLC cells, which express α_v_β_6_, were injected into NOD/SCID mice to form subcutaneous H2009 xenografts. At day 18, once the mice had formed palpable tumors, they were treated with HEPES buffered saline (HBS) as a control; free, non-liposome encapsulated, doxorubicin; α_v_β_6_-targeted 1.3% H2009.1 tetrameric liposomal doxorubicin; or control liposomal doxorubicin formulations. Two different liposome formulations were used as non-targeted controls: 1.3% “naked”, no peptide, liposomes and 1.3% scH2009.1 tetrameric liposomes. The 1.3% naked liposomes serve as a normal, non-peptide targeted liposome control, which should only accumulate passively in tumors based on the EPR effect. The 1.3% scH2009.1 tetrameric liposomes display a sequence scrambled version of the H2009.1 peptide that does not target α_v_β_6_; therefore, these liposomes serve as a control for the specificity of the H2009.1 peptide and assure that the charge and hydrophilicity of the peptide are not driving tumor accumulation. The mice were treated via tail vein intravenous injections once weekly for 3 weeks with 4 mg/kg of each liposome formulation, based on total doxorubicin concentration, on days 18, 25, and 32 after tumor cell implantation. This regimen will provide the maximum tolerated lifetime dose for NOD/SCID mice over a period of 2 weeks [56]. Weekly injections were chosen based on previous studies; more injections at a lower dose have proven to be of similar efficacy as a higher weekly dose for other liposomes with the same lipid formulation [2,16,57,58].

**Figure 1:**
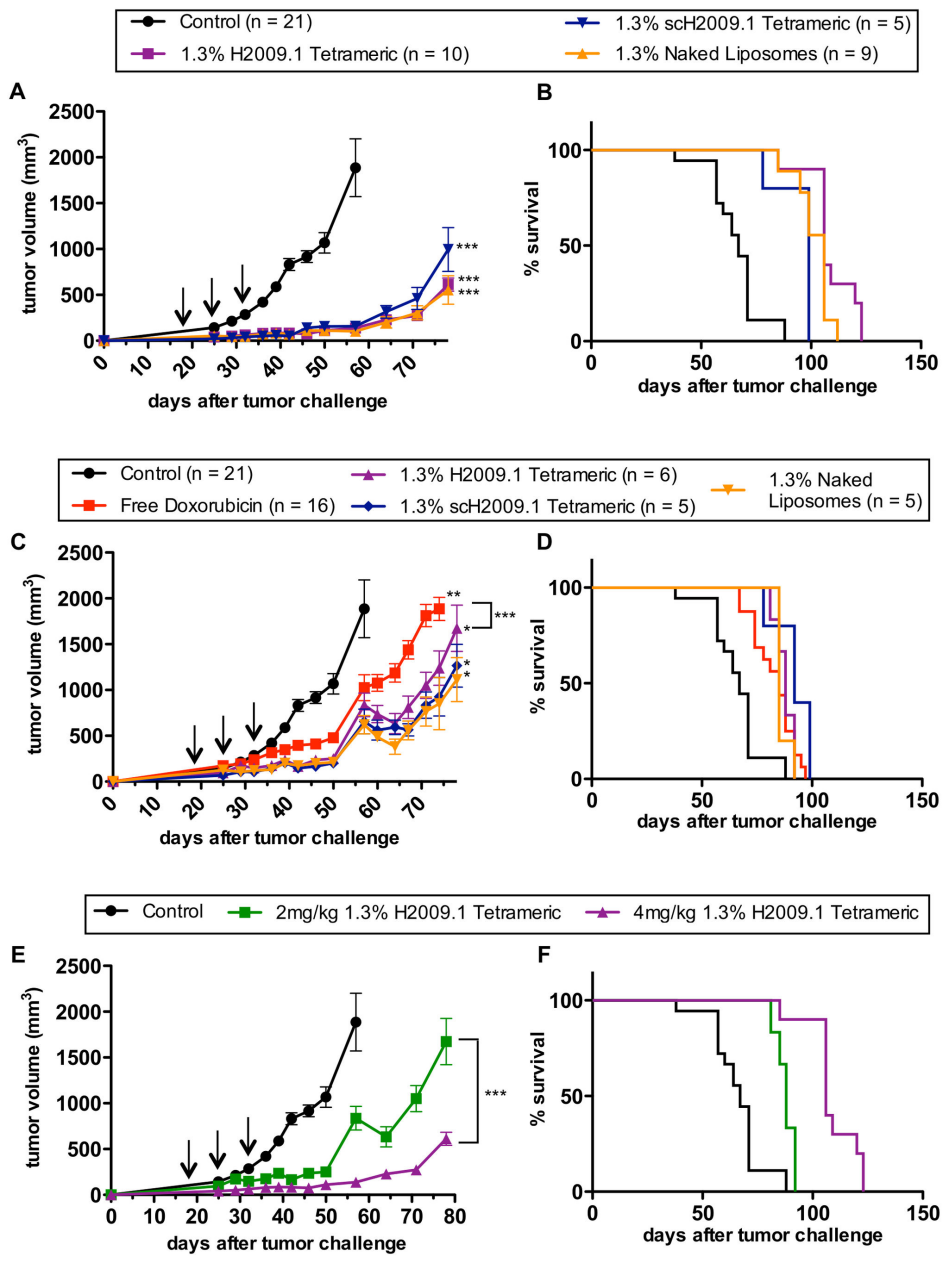
The 1.3% H2009.1 tetrameric liposomes inhibit tumor growth to the same extent as control liposomes. Subcutaneous H2009 tumors were established in the flank of NOD/SCID mice. Tumor-bearing mice were treated with HBS (control) and either 4mg/kg (*A*–*B*) or 2mg/kg (*C*–*D*) of free doxorubicin or the 1.3% H2009.1 tetrameric, scH2009.1 tetrameric, or naked liposomes, based on the total concentration of doxorubicin. *A*) Tumor growth curves for mice treated with 4mg/kg of the different liposome formulations demonstrate that the α_v_β_6_-targeting 1.3% H2009.1 tetrameric liposomes inhibit tumor growth to the same extent as the control scH2009.1 tetrameric and naked, no peptide, liposomes. (*B*) Kaplan-Meier survival curves for mice treated with 4mg/kg of the different liposome formulations demonstrate that all of the liposome treatments significantly increase survival compared to control mice (p < 0.0001 for the H2009.1 tetrameric and naked liposomes and p = 0.0007 for the scH2009.1 tetrameric liposomes), and treatment with the H2009.1 tetrameric liposomes increases survival compared to treatment with control scH2009.1 tetrameric liposomes (p = 0.0068). (*C*) Tumor growth curves for mice treated with 2mg/kg of the different liposome formulations demonstrate that free doxorubicin and all of the liposome formulations significantly inhibit tumor growth compared to control mice, and all 3 liposome formulations inhibit tumor growth to the same extent at levels significantly better than free doxorubicin. (*D*) Kaplan-Meier survival curves for mice treated with 2mg/kg of the different liposome formulations demonstrate no significant survival difference between mice treated with either free doxorubicin or any of the liposome formulations. (*E* & *F*) Direct comparisons of the tumor growth (*E*) and survival (*F*) of mice treated with 4mg/kg or 2mg/kg of the 1.3% H2009.1 tetrameric liposomes demonstrates that the 4mg/kg treatment was significantly better at inhibiting tumor growth and significantly increased survival (p = 0.0005). Arrows indicate treatment days. *p < 0.05, ** p < 0.01, *** p < 0.001verses control, unless otherwise indicated with brackets.

As depicted in Figure 1A, all liposome formulations significantly inhibited tumor growth compared to the control HBS treated group, with p values less than 0.001. However, no difference was observed between any of the liposome formulations. The targeted 1.3% H2009.1 tetrameric liposomes inhibited tumor growth to the same extent as both the control scH2009.1 tetrameric or naked liposomes. The tumor growth rates are virtually identical up to day 64, at which point the scH2009.1 tetrameric curve separates out slightly but is still within the margin of error of the H2009.1 tetrameric and naked liposome groups. Importantly, this effect is reproducible; the data in Figure 1A represent the combination of multiple separately conducted experiments.

Overall survival of the mice on the different treatment regimen was also determined. Free, non-liposome encapsulated, doxorubicin proved toxic to the mice at this 4 mg/kg dosing regimen. The free doxorubicin treated mice all died on day 32, before receiving the third and final dose of drug. Therefore, two injections of 4 mg/kg doxorubicin, for a total of 8 mg/kg doxorubicin, are sufficient for drug-induced toxicity in our studies. All of the other mice lived until they were sacrificed due to their tumors reaching a length of 2 cm (Figure 1B). As expected from the tumor growth curves, there was a significant survival difference between the control group and all of the liposome treatment groups (p < 0.0001 for the 1.3% H2009.1 tetrameric and naked liposomes and p = 0.0007 for the 1.3% scH2009.1 tetrameric liposomes). While three of the 1.3% H2009.1 tetrameric liposome treated mice lived longer than any of the 1.3% naked liposome treated mice, these differences were not statistically significant. However, there was a significant survival difference between mice treated with the 1.3% H2009.1 tetrameric and scH2009.1 tetrameric liposomes (p = 0.0068). Thus, although treatment with the 1.3% H2009.1 tetrameric liposomes did not improve therapeutic efficacy at a 4 mg/kg dosing regimen compared to the non-targeted naked liposomes, these α_v_β_6_-targeted liposomes increased survival compared to the control peptide scH2009.1 tetrameric liposomes.

### Effects of Liposome Dosage on the In Vivo Efficacy of the H2009.1 Peptide Liposomes

As there was no increased therapeutic benefit for 1.3% liposomes displaying the H2009.1 tetrameric peptide verses control naked liposomes, we reasoned that the α_v_β_6_-specific targeting of the H2009.1 peptide liposomes might be masked by the efficacy of the naked liposomes at treatment concentrations of 4 mg/kg (12 mg/kg total). This could result by saturating the receptor such that differences between active and passive targeting are diminished. Alternatively, there may be a critical threshold for drug efficacy above which delivery of more doxorubicin does not increase cell death. Therefore, we decreased the treatment concentrations by 2-fold. As before, H2009 xenografts were established and treatment begun at day 18 with either HBS, free doxorubicin, 1.3% H2009.1 tetrameric liposomal doxorubicin, 1.3% scH2009.1 tetrameric liposomal doxorubicin, or 1.3% naked liposomal doxorubicin. This time, however, the mice were treated with 2 mg/kg instead of 4 mg/kg of drug at days 18, 25, and 32. It is important to note that this represents a 2-fold reduction in total doxorubicin dosage and in the total number of liposomes administered. Treatment at these lower drug concentrations did not increase the efficacy of the H2009.1 tetrameric liposomes compared to naked, non-targeted, liposomes. Once again, there was no difference in tumor growth between mice treated with the 1.3% H2009.1 tetrameric, scH2009.1 tetrameric, and naked liposomes (Figure 1C). All of the liposome formulations and free doxorubicin inhibited tumor growth compared to the control HBS-treated mice (p < 0.05 and p < 0.01, respectively), and all 3 liposome formulations inhibited tumor growth compared to free doxorubicin (p < 0.001). However, the 3 liposome formulations themselves were indistinguishable.

Similarly, while the H2009.1 targeted liposomes increased survival compared to control mice (p = 0.0025), there was no survival difference between mice treated with the 3 liposome formulations (Figure 1D). While the liposome formulations slow tumor growth compared to 2 mg/kg of free doxorubicin, there is no significant difference in overall survival. Directly comparing the tumor sizes of mice treated with 2 mg/kg verses 4 mg/kg of 1.3% H2009.1 tetrameric liposomal doxorubicin (Figure 1E), demonstrates that the higher dose 4 mg/kg treatments were significantly more effective at inhibiting tumor growth than the lower dose 2 mg/kg treatments (p < 0.001) and also increased survival (Figure 1F, p = 0.0005). In a similar fashion, animals were treated with 6 mg/kg of 1.3% H2009.1 tetrameric liposomal doxorubicin. The 6 mg/kg dosing regimen proved toxic to mice. Within a group of 5 animals, 2 died after the second injection with a 6 mg/kg dose while a third animal died immediately upon the third injection. For the remaining two animals, tumor growth rate was the same as that observed for the 4mg/kg treatment. Therefore, although the 1.3% H2009.1 tetrameric liposomes inhibit H2009 tumor growth to the same extent as the non-targeted naked liposomes at both low and high drug treatment concentrations, the higher drug treatments leads to a better therapeutic outcome.

### Effects of Peptide Affinity and Density on In Vivo Efficacy of H2009.1 Peptide Liposomes

Peptide valency altered liposomal targeting *in vitro*. Liposomes displaying the higher affinity tetrameric H2009.1 peptide were 6-fold more toxic towards α_v_β_6_-positive H2009 cells *in vitro* than liposomes displaying the lower affinity monomeric H2009.1 peptide. This is consistent with the dramatic enhancement in affinity observed upon tetramerization of the H2009.1 peptide. However, based on the failure of the H2009.1 tetrameric peptide liposomes to alter tumor size or survival compared to the non-peptide naked liposomes *in vivo*, we reasoned that a high affinity ligand might inhibit nanoparticle targeting *in vivo*, an effect often referred to as the affinity barrier. The high affinity tetrameric peptide might not penetrate the tumor as well as a lower affinity ligand due to its stronger affinity for α_v_β_6_. This could result in the tetrameric peptide liposomes accumulating only in α_v_β_6_-expressing tumor cells near the blood vessels through which they entered the tumor, without any penetration into other areas of the tumor. Alternatively, the lower affinity monomeric peptide liposomes might display better tumor distribution and better therapeutic effects by bypassing some of the α_v_β_6_-expressing tumor cells near the blood vessels to enter α_v_β_6_-expressing cells further into the tumor.

**Figure 2:**
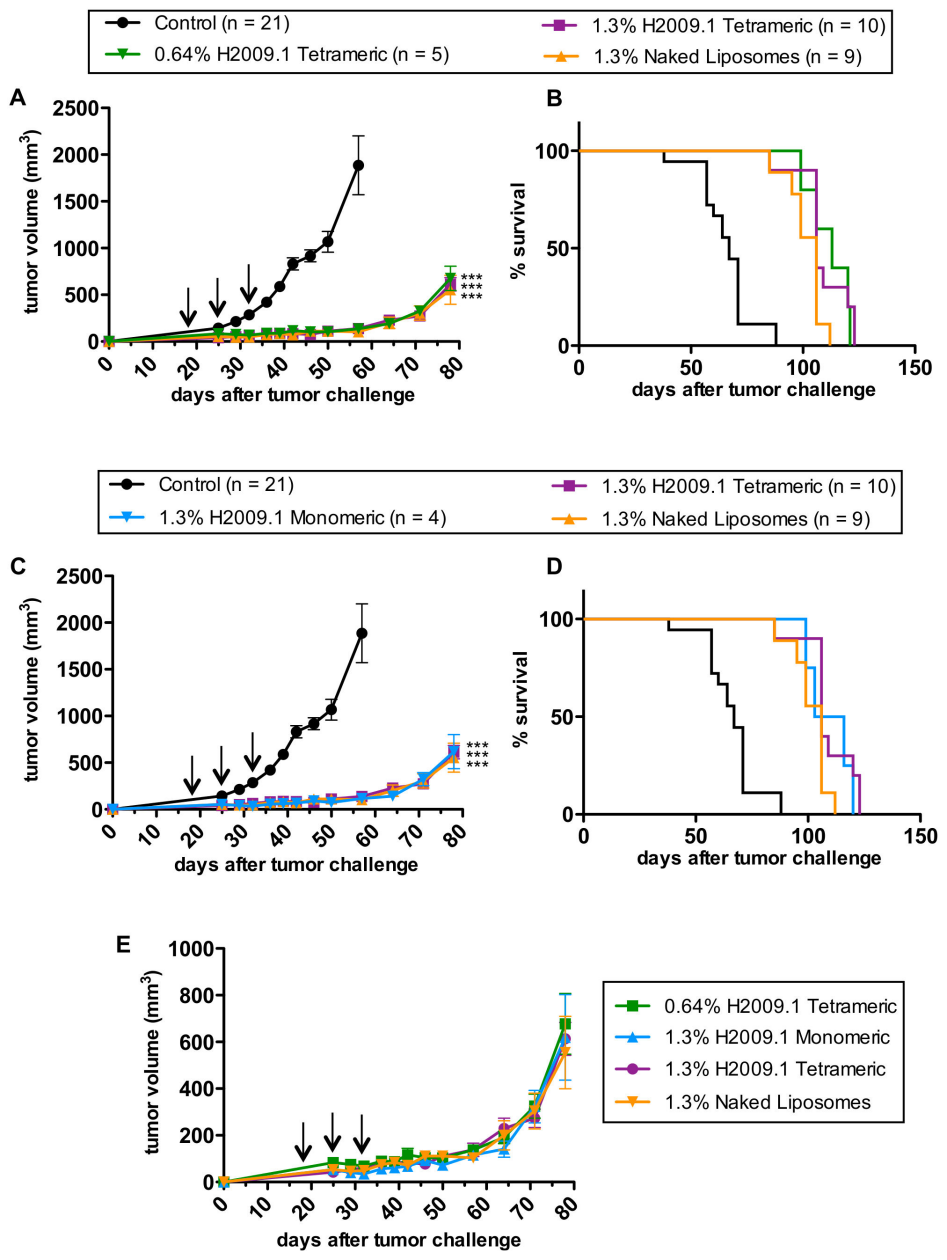
Liposomal H2009.1 peptide affinity and density do not alter *in vivo* efficacy. Subcutaneous H2009 tumors were established in the flank of NOD/SCID mice. Tumor-bearing mice were treated with HBS (control) and 4mg/kg of different liposome formulations, based on the total concentration of doxorubicin. (*A*) Tumor growth curves and (*B*) Kaplan-Meier survival curves for mice treated with 0.64% H2009.1 tetrameric, 1.3% H2009.1 tetrameric, or 1.3% naked liposomes demonstrate that changes in liposomal peptide concentration do not alter tumor growth inhibition or survival; all 3 liposome formulations inhibit tumor growth to the same extent and increase survival compared to control mice to the same extent (p < 0.0001). (*C*) Tumor growth curves and (*B*) Kaplan-Meier survival curves for mice treated with 1.3% H2009.1 monomeric, 1.3% H2009.1 tetrameric, or 1.3% naked liposomes demonstrate that changing the affinityof the peptides displayed on liposomes does not alter tumor growth inhibition or survival; all 3 liposome formulations inhibit tumor growth to the same extent and increase survival compared to control mice to the same extent (p < 0.0001). (*E*) Graphing the tumor growth curves for the 3 different H2009.1 liposome formulations and the naked liposomes on the same axis without the control group allows for a clearer visualization of the overlapping nature of the 4 different liposome curves and demonstrates that liposomal H2009.1 peptide concentration and affinity and even the presence of the H2009.1 peptide, do not affect liposomal tumor inhibition. Arrows indicate treatment days. *** p < 0.001verses control.

To test this hypothesis, we treated H2009 xenograft-bearing mice with 1.3% H2009.1 monomeric liposomal doxorubicin and compared the efficacy of this formulation to that of the 1.3% H2009.1 tetrameric liposomal doxorubicin. As demonstrated in Figure 2C-D, the valency of the H2009.1 peptide did not affect either tumor growth or the survival of the treated mice. The 1.3% H2009.1 monomeric liposomes inhibited tumor growth to the same extent as both the 1.3% H2009.1 tetrameric liposomes and the control 1.3% naked liposomes.

Another approach to modulating liposome affinity for α_v_β_6_-expressing cells is to manipulate the peptide density on the liposome surface. The concentration of tetrameric peptide displayed on the liposome surface contributes to the targeting specificity and toxicity of the H2009.1-targeted liposomes *in vitro* [39]. The 1.3% liposomes, which display approximately 1400 tetrameric peptides per liposome, led to better cell uptake and cell toxicity *in vitro* than 0.64% liposomes displaying only 700 peptides per liposome.

We treated mice bearing subcutaneous H2009 tumors with the lower peptide density 0.64% H2009.1 tetrameric liposomes and compared the efficacy of this formulation to the higher peptide density 1.3% H2009.1 tetrameric liposomes. These experiments were performed as before, treating once weekly for 3 weeks starting at day 18 using the 4 mg/kg dose that led to better efficacy. Unlike the *in vitro* experiments, which resulted in a 3-4 fold targeting increase and a 1.5-fold toxicity increase for the 1.3% H2009.1 tetrameric liposomes verses the 0.64% H2009.1 tetrameric liposomes [39], there was no efficacy difference between the two formulations against H2009 xenografts (Figure 2A-B). Both the tumor growth curves and survival curves were similar for the liposomes displaying different amounts of peptide. Thus, while H2009.1 peptide concentration alters liposomal targeting *in vitro*, this does not hold true *in vivo*. Figure 2E shows the 0.64% H2009.1 tetrameric, 1.3% H2009.1 monomeric, 1.3% H2009.1 tetrameric, and 1.3% naked liposomes graphed on the same axis. Unlike the *in vitro* studies, which revealed better targeting for all of the H2009.1 peptide liposomes compared to the naked liposomes, with the 1.3% H2009.1 tetrameric liposomes displaying the best efficacy, there was no therapeutic difference between any of the liposome formulations *in vivo*.

### Biodistribution and Tumor Accumulation of Different Liposome Formulations

Based on the failure of the α_v_β_6_-specific H2009.1 tetrameric liposomes to treat α_v_β_6_-positive tumors more effectively *in vivo* than naked, no peptide, liposomes, we examined the biodistribution of the different liposome formulations and the total levels of liposome accumulation in the tumors. It is important to distinguish whether the H2009.1 tetrameric peptide drives higher liposome accumulation in the tumors for the peptide bearing liposomes or whether the liposome nanoparticle itself drives tumor accumulation based on the EPR effect. If the EPR effect is driving liposome accumulation in tumors, we expect to see the same level of tumor accumulation for the H2009.1-peptide targeted liposomes as the naked liposomes. To visualize and quantify liposome biodistribution and tumor uptake, the liposomes were prepared as before except that they were not loaded with doxorubicin and were instead labeled with the lipophilic near infrared dye DiR, which incorporates into the lipid membrane. As before, 1.3% liposomes bearing the H2009.1 tetrameric, scH2009.1 tetrameric, or no peptide were prepared.

**Figure 3:**
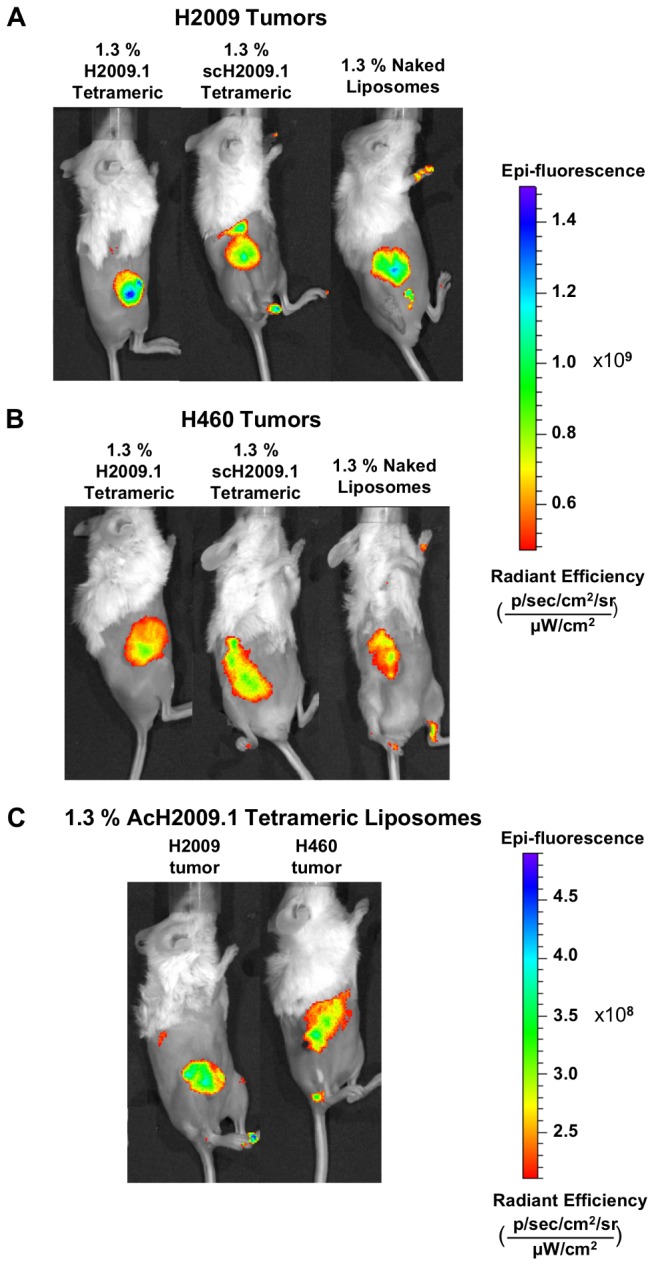
Targeted H2009.1 and control liposomes accumulate in tumors to the same extent. Subcutaneous α_v_β_6_-positive H2009 (panel A) or α_v_β_6_-negative H460 tumors (panel B) were established in the right flank of NOD/SCID mice. Tumor bearing mice were injected via tail vein with either 1.3% H2009.1 tetrameric, AcH2009.1 tetrameric, scH2009.1 tetrameric, or naked liposomes labeled with the near infrared dye DiR. Animals were imaged at 24, 48, and 72 hours post-liposome injection. Shown are representative images from the 72 hour time point. Despite the α_v_β_6_-targeting abilities of the H2009.1 peptide, all liposomes, except for the AcH2009.1 tetrameric liposomes, accumulated at similar levels in α_v_β_6_-positive H2009 tumors and α_v_β_6_-negative H460 tumors. (*C*) 1.3% AcH2009.1 tetrameric liposome accumulation in both H2009 and H460 tumors. Note the difference in the epi-fluorescence scale compared to panels A and B.

In order to fully examine H2009.1 peptide-driven liposome targeting to α_v_β_6_, two different tumor models were examined for liposome accumulation: α_v_β_6_-positive H2009 tumors and α_v_β_6_-negative H460 tumors. Xenograft tumors were established in the right flank of NOD/SCID mice, and tumor bearing mice were injected via tail vein with the different DiR-labeled liposome formulations for imaging via whole mouse fluorescent imaging at 24, 48, and 72 hours post-injection. Table 1 lists quantified values of liposome accumulation in both H2009 and H460 tumors, and Figure 3 shows representative images for one mouse from each liposome formulation at 72 hours post-injection. As is evident in Figure 3A, there was no difference in liposome accumulation in H2009 tumors for the H2009.1 tetrameric, scH2009.1 tetrameric or naked liposomes. While each liposome formulation continued to accumulate in the tumors up to 72 hours (Table 1), the H2009.1 tetrameric liposomes did not increase targeting to the α_v_β_6_-expressing H2009 tumors.

**Table 1:** Quantification of tumor accumulation of different liposome formulations as determined by whole mouse fluorescent imaging.

	**Liposome Formulation**	**Radiant Efficiency (x 10^9^)**		
		**24hrs**	**48hrs**	**72hrs**
**H2009 tumors**	H2009.1 Tetrameric	10.1 ± 0.0922	10.2 ± 0.601	12.2 ± 0.452
	AcH2009.1 Tetrameric	5.96 ± 1.86	5.36 ± 1.91	5.96 ± 1.59
	scH2009.1 Tetrameric	9.91 ± 1.37	12.1 ± 0.676	12.8 ± 1.03
	Naked	9.41 ± 1.65	11.3 ± 1.54	13.3 ± 2.32
**H460 tumors**	H2009.1 Tetrameric	12.4 ± 1.90	12.8 ± 1.80	12.6 ± 1.39
	AcH2009.1 Tetrameric	5.30 ± 0.529	4.95 ± 0.327	5.08 ± 0.373
	scH2009.1 Tetrameric	12.3 ± 3.01	14.1 ± 4.68	14.9 ± 3.77
	Naked	8.26 ± 1.20	11.3 ± 1.65	11.2 ± 0.840

Quantification of the tumor accumulation of DiR-labeled liposomes in either α_v_β_6_-positive H2009 or α_v_β_6_-negative H460 tumors at different time points post-liposome injection, as visualized by whole mouse fluorescent imaging in Figure 3.

Analogous to the α_v_β_6_-positive H2009 tumors, there was no difference in liposome accumulation between the different liposome formulations in the α_v_β_6_-negative H460 tumors (Table 1 & Figure 3B). These results are not surprising as H460 tumors do not express α_v_β_6_ and therefore should not accumulate higher levels of α_v_β_6_-targeted H2009.1 tetrameric liposomes compared to the control liposome formulations. However, the results with the α_v_β_6_-positive H2009 tumors suggest that the tumor accumulation of the H2009.1 tetrameric, scH2009.1 tetrameric, and naked liposomes is driven by the EPR effect. Regardless of the α_v_β_6_ expression levels of the tumor or the ability of the liposomes to specifically target α_v_β_6_, all liposome formulations accumulate to the same extent in the H2009 and H460 tumors.

**Figure 4:**
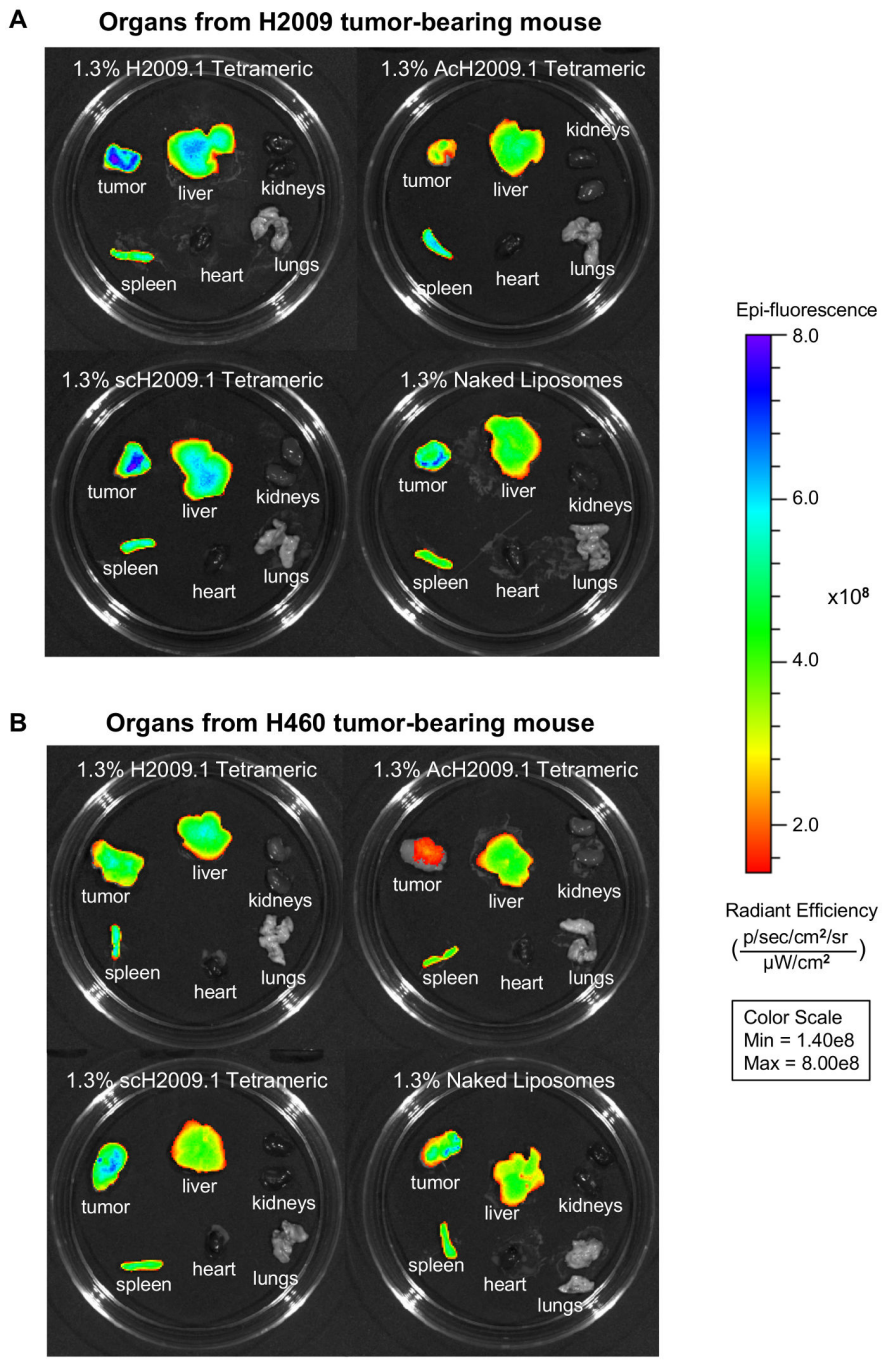
Targeted H2009.1 and control liposomes accumulate in tumors and clear through the liver and spleen. Subcutaneous α_v_β_6_-positive H2009 or α_v_β_6_-negative H460 tumors were established in the right flank of NOD/SCID mice. Tumor bearing mice were injected via tail vein with either 1.3% H2009.1 tetrameric, AcH2009.1 tetrameric, scH2009.1 tetrameric, or naked liposomes labeled with the near infrared dye DiR. At 72 hours post-injection, the mice were sacrificed and the tumors and organs removed for *ex vivo* fluorescent imaging. (*A*) Representative images of liposome accumulation in tumors and organs from α_v_β_6_-positive H2009 tumor bearing mice. Like the *in vivo* imaging in Figure 3, despite the α_v_β_6_-targeting abilities of the H2009.1 peptide, all liposome formulations except for the AcH2009.1 tetrameric liposomes accumulated in tumors to the same extent. (*B*) Representative images of liposome accumulation in tumors and organs from α_v_β_6_-negative H460 tumor bearing mice. Similar to the *in vivo* imaging in Figure 3, the H2009.1 tetrameric and scH2009.1 tetrameric liposomes accumulated in tumors to the same extent, with the AcH2009.1 and naked liposomes accumulating in tumors at levels that are 2-fold lower.

At 72 hours, the mice in each liposome group were sacrificed and their organs removed for *ex vivo* fluorescent imaging to examine the biodistribution of the liposomes in the different tumor-bearing mice. Figure 4 shows representative images of the organs, and Table 2 lists the quantification of the liposome accumulation in the organs and tumors imaged *ex vivo*. As expected for a liposome nanoparticle, all of the liposome formulations appear to clear through the liver and spleen. No liposome accumulation was observed in the heart, kidneys, or lung for all formulations. Like the *in vivo* imaging, *ex vivo* imaging of organs and tumors from mice bearing H2009 tumors demonstrated no difference in tumor accumulation among the different liposome formulations.

**Table 2:** Quantification of tumor and organ accumulation of different liposome formulations as determined by *ex vivo* fluorescent imaging.

	**Liposome Formulation**	**Tumor or Organ**	**Radiant Efficiency (x 10^9^)**
**H2009 tumors**	H2009.1 Tetrameric	Tumor	5.25 ± 0.200
		Liver	13.0 ± 1.01
		Spleen	2.59 ± 0.341
	AcH2009.1 Tetrameric	Tumor	2.29 ± 0.263
		Liver	9.17 ± 0.420
		Spleen	2.04 ± 0.196
	scH2009.1 Tetrameric	Tumor	5.27 ± 0.465
		Liver	15.2 ± 3.21
		Spleen	3.37 ± 0.949
	Naked	Tumor	6.35 ± 1.64
		Liver	11.0 ± 1.03
		Spleen	1.73 ± 0.117
**H460 tumors**	H2009.1 Tetrameric	Tumor	7.14 ± 0.374
		Liver	10.7 ± 1.46
		Spleen	1.71 ± 0.132
	AcH2009.1 Tetrameric	Tumor	4.88 ± 3.20
		Liver	21.6 ± 13.3
		Spleen	3.27 ± 1.90
	scH2009.1 Tetrameric	Tumor	6.36 ± 0.935
		Liver	8.06 ± 0.542
		Spleen	1.63 ± 0.247
	Naked	Tumor	3.77 ± 0.404
		Liver	8.33 ± 1.28
		Spleen	1.53 ± 0.154

Quantification of the organ and tumor accumulation of DiR-labeled liposomes after animal sacrifice at 72 hours post-liposome injection, as visualized by *ex vivo* fluorescent imaging in Figure 4.

Conversely, *ex vivo* imaging of the organs and tumors from H460-tumor bearing mice gave slightly different results than the *in vivo* tumor imaging. While the H2009.1 tetrameric and scH2009.1 tetrameric liposomes accumulated at equal levels, the naked liposomes appeared to accumulate at levels 2-fold lower. It is unclear why this discrepancy appeared between the *in vivo* and *ex vivo* imaging. As the H460 tumors do not express α_v_β_6_, they should not specifically accumulate the α_v_β_6_-targeting H2009.1 tetrameric liposomes. Additionally, dye-labeled H2009.1 tetrameric peptide homes to H2009 tumors and not H460 tumors (data not shown). However, nonspecific accumulation based on peptide charge may play a larger role in peptide functionalized-liposome delivery to H460 tumors. Each H2009.1 and scH2009.1 tetrameric peptide-bearing liposome has a +8 charge per peptide, with 1,400 peptides per liposome. As *ex vivo* imaging allows for imaging of the entire tumor, such nonspecific accumulation may be more evident in *ex vivo* as opposed to *in vivo* imaging.

While the H2009.1 tetrameric peptide does not alter liposome accumulation in the α_v_β_6_-positive H2009 tumors, this could be an effect of the tumor vasculature structure of that particular tumor type. A different tumor model with its own unique tumor vasculature pattern might experience different levels of liposome accumulation based on the number or leakiness of blood vessels; this could allow the H2009.1 peptide to override the EPR effect and produce a greater degree of liposome extravasation into the tumor tissue. Therefore, we also examined the accumulation of different liposome formulations in mice bearing α_v_β_6_-positive H1975 tumors. As with the H2009 and H460 tumor models, this experiment was performed by injecting DiR dye-labeled liposomes in the tail vein of H1975 tumor-bearing mice and then imaging the mice at 24, 48, and 72 hours after liposome injection. Imaging the liposome-injected H1975 tumor-bearing mice gave results similar to those of the H2009 tumor-bearing mice (Figure S1, Table S1). Once again, there was no difference between the tumor accumulation of the H2009.1 tetrameric, scH2009.1 tetrameric, and naked liposomes. These same results also held true for *ex vivo* imaging of the organs and tumors from the H1975 tumor-bearing mice (Figure S1, Table S2). Therefore, changing to a different α_v_β_6_-expressing tumor model did not alter the EPR-driven accumulation of all of the liposome formulations.

### Biodistribution and Tumor Accumulation of an Acetylated H2009.1 Liposome

One possible reason for the failure of the H2009.1-liposomes to accumulate at higher levels than control liposomes in α_v_β_6_-expressing tumors may stem from the H2009.1 peptide stability *in vivo*. This is especially critical given the long *in vivo* half-life of pegylated liposomes. The half-life of liposomal doxorubicin is > 18 hours in mice and > 50 hours in humans [2], while the half-life of the H2009.1 tetrameric peptide in human serum is 4 hours (unpublished data). Thus, the H2009.1 peptide may begin to degrade while still conjugated to a circulating liposome, reducing the ability of the peptide to specifically target liposomes at later time points. Acetylation of the amino-terminus is a common method to increase *in vivo* stability of peptides by preventing N-terminal peptide degradation by peptidases and proteases. As the RGDLXXL-binding domain of the peptide is at the N-terminus of the peptide, serum peptidases may clip the peptide rendering it inactive. N-terminal acetylation could reduce this problem.

Liposomes were conjugated to an amino-terminus acetylated version of the H2009.1 tetrameric peptide, labeled as AcH2009.1 tetrameric. Acetylation of the H2009.1 peptide does not affect its affinity for α_v_β_6_ and extends its half-life in human serum to >48 h. *In vivo* biodistribution of the free H2009.1 peptide showed similar tumor accumulation for the parental and acetylated versions of H2009.1. However, acetylation decreased nonspecific uptake of the free peptide in other organs (data not shown). Surprisingly, acetylation of the peptide reduced tumor accumulation in the context of liposomal display; AcH2009.1 tetrameric liposomes targeted the H2009 tumors ~2-fold less than any of the other liposome formulations in both the H2009 and H460 tumors (Figure 4, Table 2). This is true for all time points. However, *ex vivo* organ imaging at 72 h does not demonstrate a significant difference in liver uptake between AcH2009.1 liposome and the other liposome formulations. It is not clear why acetylation has this effect as the peptide affinity remains unchanged. Acetylation reduces the charge of each tetrameric peptide by +4, and this reduction in charge may play a role in the difference in tumor accumulation. Additionally, without full pharmacokinetic studies, we cannot rule out the possibility that acetylation of the peptide decreases the *in vivo* half-life of the liposomes.

### Penetration of Liposomes into Tumor Tissue

While the H2009.1 tetrameric, scH2009.1 tetrameric, and naked liposomes accumulate in tumors to the same extent, it is unclear whether they display a similar tumor distribution or whether they differ in terms of penetration and location within the tumor tissue. In order to achieve effective α_v_β_6_ targeting, the H2009.1 tetrameric liposomes must enter the tumor through blood vessels and then penetrate into the tumor tissue. To fully understand why the H2009.1 tetrameric liposomes do not increase therapeutic effects compared to the scH2009.1 tetrameric or naked liposomes, we investigated the tumor penetration and localization of the different liposome formulations. Liposomes were prepared with the fluorescent lipophilic dye DiI, which incorporates into the lipid membrane. These liposomes are similar to the liposomes prepared for the whole mouse imaging studies except that they include a lower wavelength dye that allows for visualization of the liposomes via microscopy of tumor sections.

**Figure 5:**
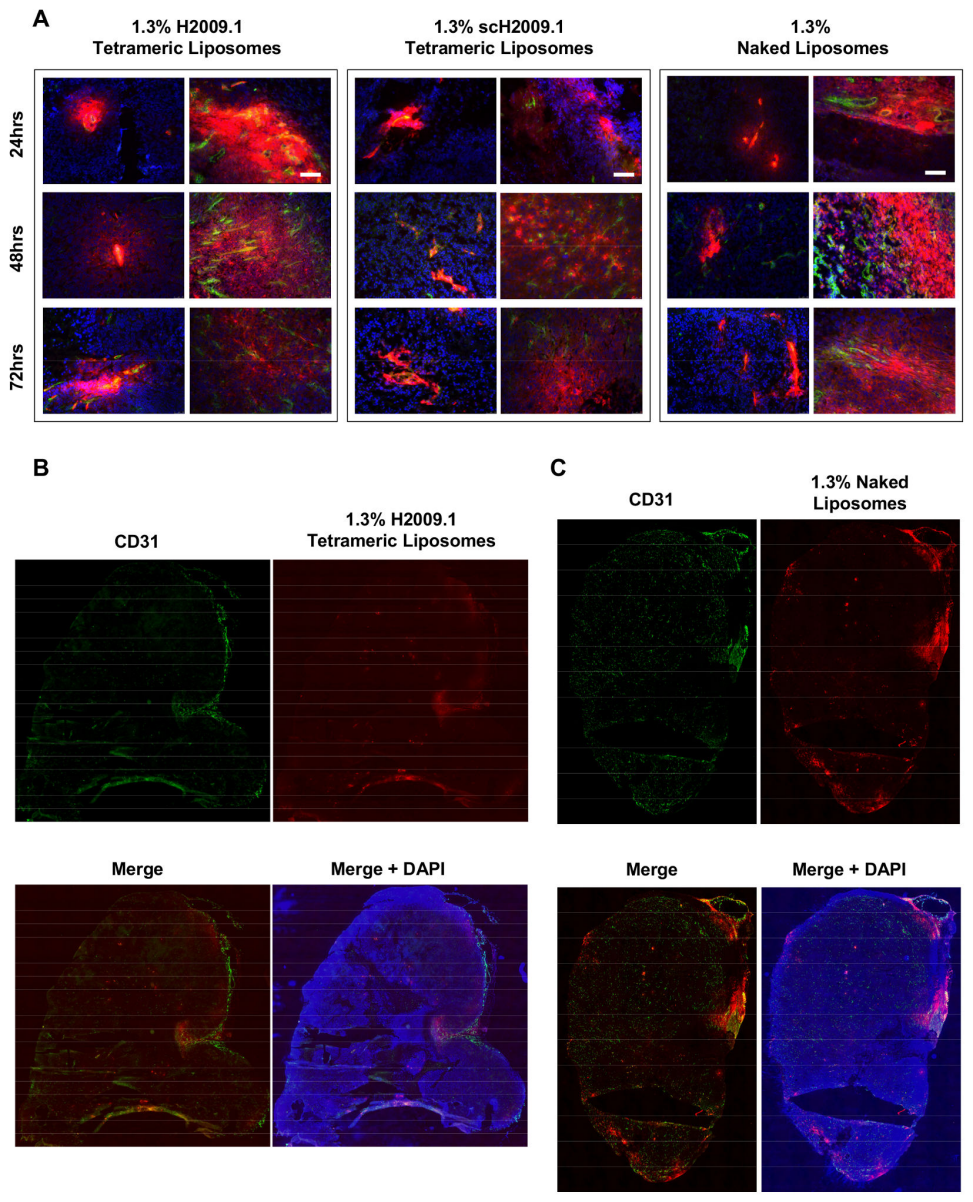
Both targeted H2009.1 and control liposomes accumulate only in the perivasculature regions of H2009 tumors. Subcutaneous α_v_β_6_-positive H2009 tumors were established in the flank of NOD/SCID mice. Tumor bearing mice were injected via tail vein with either 1.3% H2009.1 tetrameric, scH2009.1 tetrameric, or naked liposomes labeled with the dye DiI. At 24, 48, or 72 hours post-liposome injection, the mice were sacrificed and the tumors removed for sectioning and fluorescent microscopy. Blue – DAPI, Red – DiI-labeled liposomes, and Green – CD31 vasculature stain. (*A*) 10X images of liposome accumulation in tumors. The white scale bars indicate 100 μm. At each time point, all liposomes are clustered in the areas immediately adjacent to the vasculature, with the same pattern of accumulation for all of the different liposome formulations. Although there are areas of high liposome accumulation, they only occur in vascular-rich areas with large blood vessels. (*B*) Representative whole tumor image from a mouse injected with 1.3% H2009.1 tetrameric liposomes and sacrificed 48 hours after injection. The liposome accumulation overlaps with the highly vascularized periphery of the tumor. (*C*) Representative whole tumor image from a mouse injected with 1.3% naked liposomes and sacrificed 48 hours after injection. Like the targeting H2009.1 tetrameric liposomes, the naked control liposomes display the same overlap with the highly vascularized periphery of the tumor.

**Figure 6:**
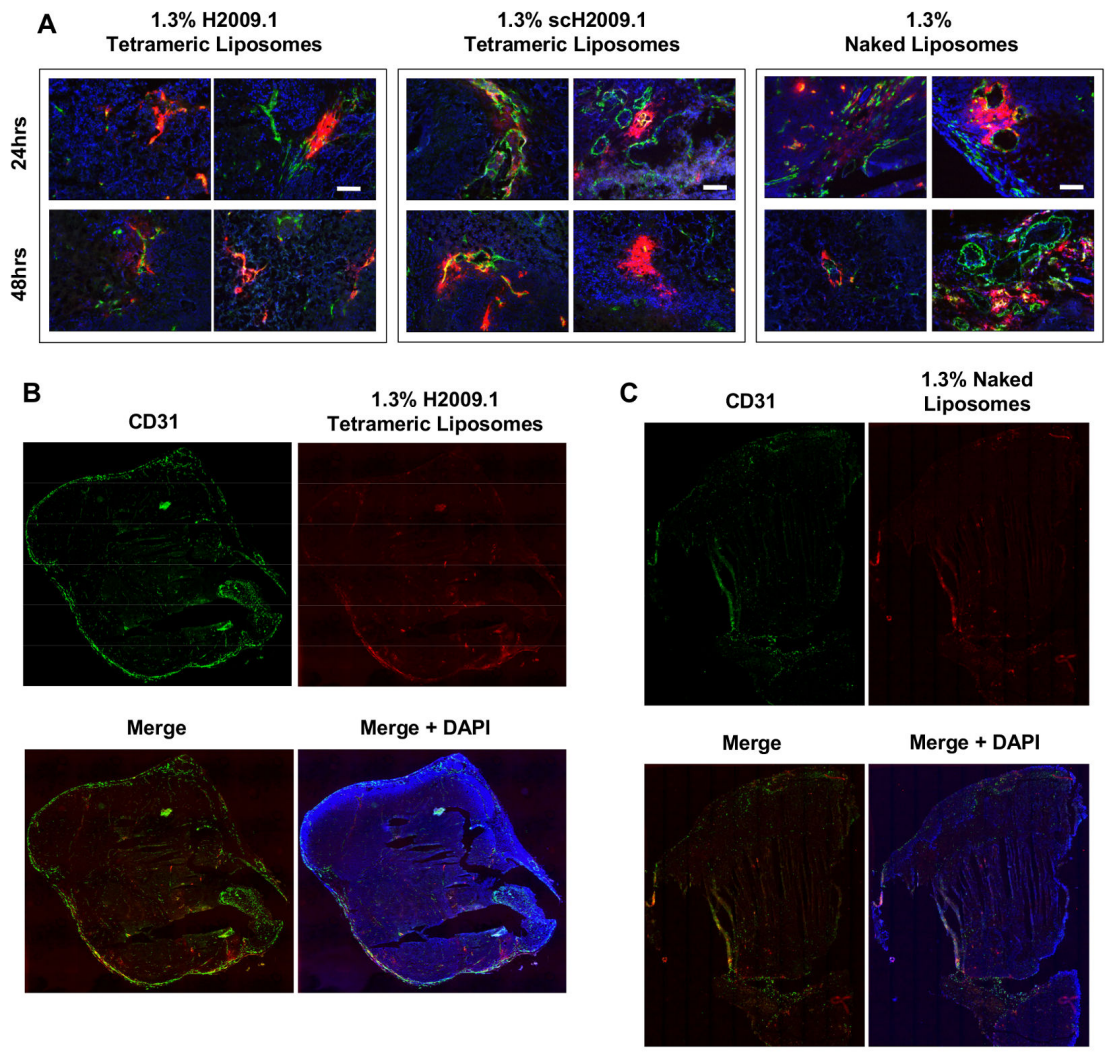
Both targeted H2009.1 and control liposomes accumulate only in the perivasculature regions of H460 tumors. Subcutaneous α_v_β_6_-negative H460 tumors were established in the flank of NOD/SCID mice. Tumor bearing mice were injected via tail vein with either 1.3% H2009.1 tetrameric, scH2009.1 tetrameric, or naked liposomes labeled with the dye DiI. At 24 or 48 hours post-liposome injection, the mice were sacrificed and the tumors removed for sectioning and fluorescent microscopy. Blue – DAPI, Red – DiI-labeled liposomes, and Green – CD31 vasculature stain. (*A*) 10X images of liposome accumulation in tumors. The white scale bars indicate 100 μm. At both time points, all liposomes are clustered in the areas immediately adjacent to the vasculature, with the same pattern of accumulation for all of the different liposome formulations. (*B*) Representative whole tumor image from a mouse injected with 1.3% H2009.1 tetrameric liposomes and sacrificed 48 hours after injection. The liposome accumulation overlaps with the highly vascularized periphery of the tumor. (*C*) Representative whole tumor image from a mouse injected with 1.3% naked liposomes and sacrificed 48 hours after injection. The naked control liposomes also overlap with the highly vascularized periphery of the tumor.

Mice bearing either α_v_β_6_-positive H2009 or H1975 or α_v_β_6_-negative H460 xenografts were injected via tail vein with DiI-labeled 1.3% H2009.1 tetrameric, scH2009.1 tetrameric, or naked liposomes and sacrificed at 24, 48, or 72 hours post-liposome injection. At the time of sacrifice, tumors were removed and frozen for sectioning and microscopy. The tumor sections were stained for tumor blood vessels using an anti-CD31 antibody, and DAPI was added to visualize nuclei. Representative images of liposome accumulation in H2009 tumors are shown in Figure 5, in H1975 tumors in Figure S2, and in H460 tumors in Figure 6. Both the α_v_β_6_-positive H2009 and H1975 tumors and the α_v_β_6_-negative H460 tumors exhibited similar patterns of liposome accumulation. All of the liposome formulations accumulated in the tumors in the perivascular areas immediately adjacent to the tumor blood vessels, and none of the liposomes penetrated further into the tumor tissue. While there were some areas of high liposome accumulation, these were all found in highly vascularized regions of the tumors; additionally, the same pattern of high liposome accumulation was observed for the H2009.1 tetrameric, scH2009.1 tetrameric, and naked liposomes in each tumor type. Liposome accumulation was mostly observed in regions with prominent blood vessels near the tumor periphery. Other areas of the tumor with either smaller or more isolated blood vessels demonstrated little liposome accumulation. Thus, the ~100 nm liposome size may prevent extravasation through smaller blood vessels.

**Figure 7:**
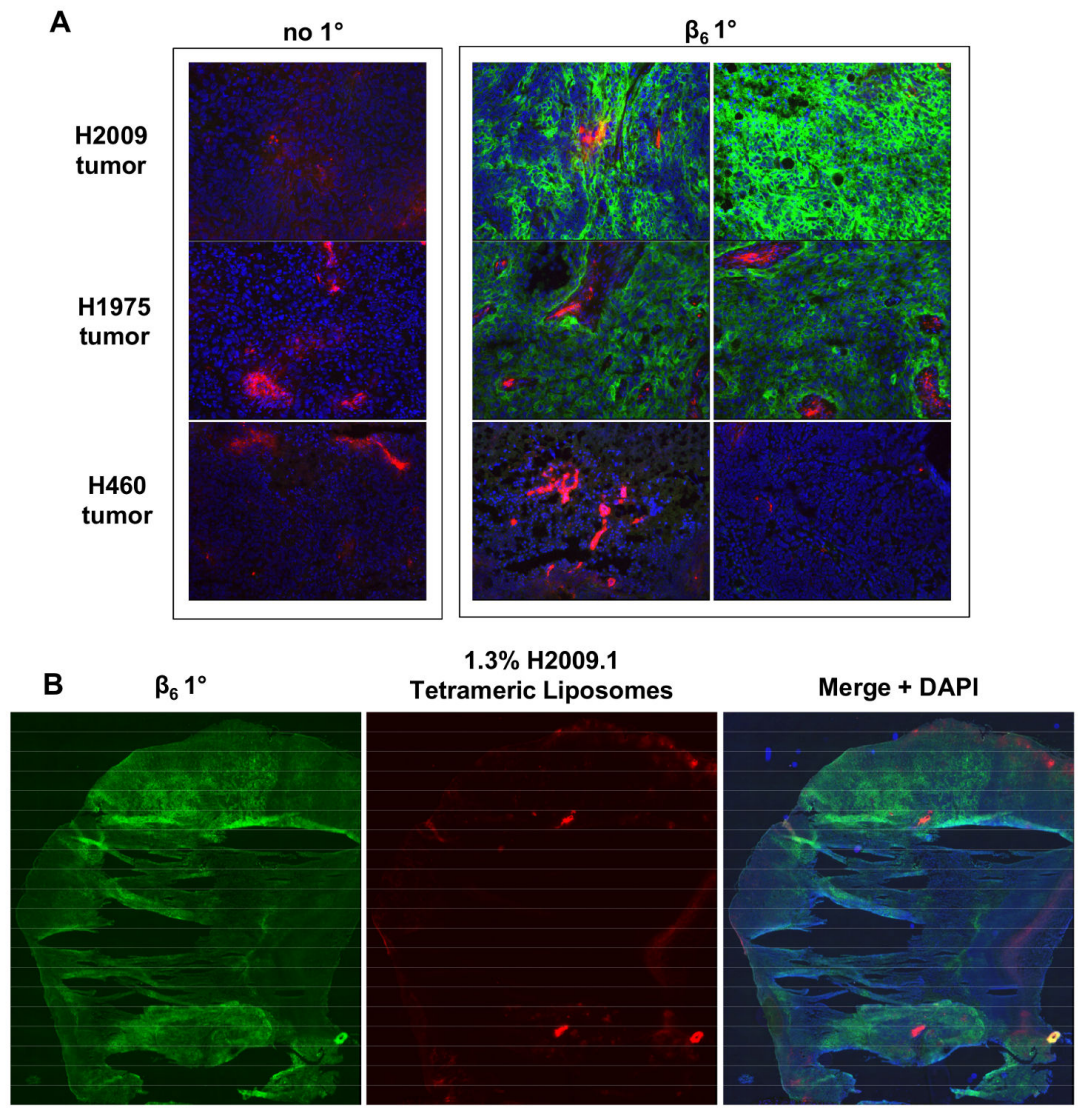
H2009 and H1975 tumors express α_v_β_6_ while H460 tumors do not express the integrin. Subcutaneous H2009, H1975, or H460 tumors were established in the flank of NOD/SCID mice. Tumor bearing mice were injected via tail vein with 1.3% H2009.1 tetrameric liposomes labeled with the dye DiI. At 24 hours post-liposome injection, the mice were sacrificed and the tumors removed for sectioning and fluorescent microscopy. Blue – DAPI, Red – DiI-labeled liposomes, and Green -β_6_. (*A*) 10X images of H2009, H1975, and H460 tumors stained without a primary antibody or with a β_6_ primary antibody along with a green fluorescent secondary antibody. The H2009 and H1975 tumors stain brightly green for the presence of β_6_, while the H460 tumor does not show any β_6_ staining. (*B*) Representative whole tumor images of a β_6_-stained H2009 tumor section demonstrate that β_6_ is expressed throughout the tumor.

To verify that the receptor for the H2009.1 tetrameric peptide was still expressed in these tumors, the H2009, H1975, and H460 tumors were stained for α_v_β_6_ expression using immunofluorescence with an anti-β_6_ antibody. As expected, the H2009 and H1975 tumors exhibited high levels of β_6_ while no β_6_ was observed in the H460 tumors (Figure 7A). Additionally, β_6_ was expressed widely throughout both the H2009 and H1975 tumors, as demonstrated by whole tumor imaging of a H2009 tumor (Figure 7B). Therefore, although the receptor for the H2009.1 tetrameric liposomes is expressed throughout the tumor, the targeted liposomes are unable to reach most of the α_v_β_6_-expressing tumor cells due to a failure to penetrate through the tumor tissue. Thus, it appears that the lack of additional efficacy for the H2009.1 tetrameric liposomes compared to the naked liposomes is at least in part a result of the inability of the targeted liposomes to penetrate into the tumor tissue to enter α_v_β_6_-expressing cells.

## Discussion

The clinical success of pegylated liposomal doxorubicin, or DOXIL®, makes it an attractive nanoparticle platform for targeted drug delivery, and a variety of targeting ligands conjugated to liposomal forms of doxorubicin demonstrate increased efficacy compared to non-targeted liposomes, either by inhibiting tumor growth or by increasing survival of tumor-bearing rodents [8–20]. Several antibody-targeted forms of liposomal doxorubicin are in clinical trials [26,27] and at least one peptide-targeted form of liposomal doxorubicin is being manufactured using GMP to ease the transition to clinical trials [29]. These targeted forms of liposomal doxorubicin rely on both passive targeting from the EPR effect and on active targeting from the specificity of the targeting ligand for tumor or tumor vasculature cells.

Encouraged by the successes met with ligand-targeted forms of liposomal doxorubicin and with the goal of optimizing the therapeutic use of phage-display library selected peptides, we previously developed targeted liposomes specific for the integrin α_v_β_6_ by conjugating the α_v_β_6_-specific H2009.1 peptide to the surface of liposomal doxorubicin [39]. These studies demonstrated that liposomes displaying the higher affinity tetrameric H2009.1 peptide at approximately 1400 peptides per liposome, a 1.3% H2009.1 tetrameric formulation, had the best α_v_β_6_-specific cell targeting and toxicity. Significantly, the ideal 1.3% H2009.1 tetrameric liposome formulation was 2-fold more toxic to cells than liposomes bearing the control scrambled scH2009.1 peptide and more than 10 times more toxic than naked, no peptide, liposomes. Based on these *in vitro* results and with the goal of developing clinically relevant therapy agents from phage-display selected peptides, we chose to study the efficacy of H2009.1 peptide liposomes *in vivo*.

We examined the *in vivo* efficacy of the 1.3% H2009.1 tetrameric liposomes compared to both control no peptide, naked, liposomes and liposomes bearing the control scH2009.1 tetrameric peptide. While all 3 liposome formulations significantly inhibited tumor growth and increased survival compared to control buffer treated mice, there was no difference in efficacy between any of the liposome formulations. All of the liposome formulations tested, whether α_v_β_6_-targeted or untargeted, and regardless of the number and affinity of conjugated peptides, inhibited tumor growth to exactly the same extent. While H2009.1 peptide affinity and concentration alter liposomal targeting *in vitro*, this did not hold true *in vivo*. Significantly, the 1.3% H2009.1 tetrameric, scH2009.1 tetrameric, and naked liposomes accumulated in tumors to the same extent, regardless of tumor α_v_β_6_ expression levels. This remains true for three different NSCLC tumor xenografts.

Despite years of research on development of ligand-targeted liposomes, the role of active targeting in liposome delivery is controversial [32–36]. Mathematical modeling of the relationship between molecular size and tumor uptake predicts that particles above 50 nm in size, whether targeted or untargeted, will accumulate in tumors to the same extent based on the EPR effect [33]. In practice, similar tumor uptake has been observed for several different ligand-targeted forms of liposomal doxorubicin [37,38]. Consistent with these observations, H2009.1 peptide-targeted and untargeted liposomes accumulated in tumors at similar levels. However, increased tumor accumulation has been reported for other targeted-liposomes [21,23]. In these cases, it is not known whether the targeting ligand improves delivery to the tumor or prevents wash out of the liposomes [59].

Several targeted liposomes have shown increased efficacy compared to non-targeted liposomes despite identical EPR-driven levels of tumor accumulation, including the antibody-targeted anti-HER2 and anti-EGFR formulations of liposomal doxorubicin [37,38]. The increased efficacy of these formulations has been attributed to a differential pattern of liposome accumulation within tumors and the internalization of the targeted liposomes into tumor cells while the non-targeted liposomes remained in the extracellular space. As doxorubicin must enter the nuclei of cells to exert its effects, ligand-directed cellular internalization of the drug could easily drive beneficial therapeutic effects. Similar effects have been observed with other targeted nanoparticles in which the targeting ligand does not affect overall tumor accumulation but increases cellular uptake compared to the nontargeted counterpart [60]. We previously demonstrated that the H2009.1 peptide triggers α_v_β_6_-mediated cellular uptake [54] and, that H2009.1 modified liposomes are internalized into α_v_β_6_-expressing cells *in vitro* [39]. While the H2009.1 peptide mediates rapid cellular uptake, unfortunately a similar increase in therapeutic efficacy for H2009.1-liposomes is not observed.

Our data suggest that the EPR effect drives liposome accumulation in tumors and that active targeting driven by the H2009.1 peptide does not effectively occur after entry into the tumor tissue. Indeed, tumor distribution of the 1.3% H2009.1 tetrameric, scH2009.1 tetrameric, and naked liposomes in both α_v_β_6_-positive and α_v_β_6_-negative tumors is poor. All liposomes displayed the same pattern of tumor accumulation and remained clustered around the tumor blood vessels with little penetration past blood vessel rich areas of the tissue, and there was very little difference in liposome accumulation and penetration in α_v_β_6_-positive verses α_v_β_6_-negative tumors. Thus, we believe that the H2009.1 tetrameric peptide was unable to both effectively target α_v_β_6_ and carry liposomes into the majority of tumor cells due to an inability of the liposomes to penetrate the tumor tissue and access most of the α_v_β_6_-expressing cells. The H2009.1 peptide is unlikely to increase therapeutic efficacy compared to non-targeted liposomes if it is not able to alter liposomal tumor distribution.

Once a liposome extravasates into the tumor, it faces numerous challenges in reaching its cellular target; high interstitial pressure, dense extracellular matrix, a complex mixture of cell types and long distance between tumor cells and the nearest vessel all work against penetration of nanoparticles into the tumor [61]. Studies have demonstrated poor tumor penetration for ~100 nm nanoparticles. A detailed comparison of the tumor accumulation and penetration of pegylated gold nanoparticles ranging in size from 20-100 nm demonstrated poor tumor penetration for the 100 nm particles, with the larger nanoparticles localizing only to the area immediately adjacent to the vasculature [62]. However, there was an inverse relationship between particle size and tumor accumulation. The 100 nm nanoparticles had the highest levels of tumor accumulation, whereas tumor accumulation of the 20 nm particles was almost 40 times lower. This is most likely due to the faster renal clearance of the smaller particle resulting in less time for the particle to extravasate into the tumor. Despite lower levels of tumor accumulation, the smaller 20 nm particles exhibited the best tumor penetration and distribution. Similar results were observed with dextrans of different molecular weights [63] and with different sized quantum dots [64]. The vastly dissimilar outcomes in efficacy enhancement between different targeted liposomes may result from differences in the tumor microenvironment [65,66]. Case in point, Kaposi sarcomas are highly vascularized with leaky vessels. Subsequently, it is one of the few cancers in which DOXIL® displays a clear clinical benefit compared to free doxorubicin.

The affinity of targeting ligands is also known to affect tumor delivery. Most studies examining affinity have been performed using antibodies but are expected to hold true for other targeting ligands. Both mathematical modeling and experimental studies have demonstrated that lower affinity antibodies experience better tissue distribution [67–69]. This “binding site barrier” was first described in a modeling study by Fujimori et al. and is based on the successful binding of a ligand to tumor cells near the site of entry into the tumor; this binding then impedes ligand distribution throughout the tumor [67]. As high affinity ligands bind readily to tumor cells, there are fewer free ligands available to penetrate further into the tumor, and the high affinity ligands remain trapped in locations near where they first entered the tumor leading to heterogeneous tumor distribution. Lower affinity ligands experience faster off rates with their receptor and can therefore dissociate prior to cellular internalization and penetrate further into the tumor tissue. In contrast to these studies, the therapeutic efficacy of liposomes displaying the lower affinity H2009.1 monomeric peptide did not differ from that of liposomes displaying the higher affinity H2009.1 tetrameric peptide. Additionally, nontargeted liposomes did not demonstrate increased tumor penetration compared to the H2009.1 targeted liposomes. Thus, our results suggest that the large nanoparticle platform, not the targeting ligand, drives the lack tumor penetration.

It is important to note that the majority of peptide-guided liposomes that improve efficacy compared to a nontargeted liposome target the tumor vasculature and not the tumor cells. As vasculature-targeting peptides reach their target cells while in the bloodstream, they are not dependent on escape from the vasculature and subsequent penetration through the tumor tissue. These liposome formulations function instead as antiangiogenic therapies.

Several strategies have been explored to improve tumor distribution and penetration of nanoparticles. These strategies can seek to affect the tumor vasculature or to directly affect tumor penetration. These include treatment with tumor necrosis factor-alpha (TNF-α) [70,71], inhibition of TGFβ-signaling [65,72], pretreatment with radiotherapy, and induction of hypothermia in the tumor [73]. Alternatively, the H2009.1 peptide can be used on smaller nanoparticles which are anticipated to penetrate the tumor tissue better than liposomes. Small ligands with high affinity are also predicted to have high accumulation and retention in tumors and better distribution throughout the tumor tissue [33]. The H2009.1 peptide can easily be modified for direct conjugation to different chemotherapeutics, and a H2009.1 tetrameric peptide-paclitaxel conjugate shows promising results *in vivo*, inhibiting tumor growth to the same extent as free paclitaxel, despite a >30-fold lower toxicity *in vitro* [74].

This study highlights the challenges of *in vivo* drug targeting. Targeted therapies that work well in *vitro* do not always maintain specific efficacy *in vivo*, even when the targeting ligand itself homes to tumors *in vivo*. Tumor targeting *in vivo* depends on many aspects not present in the *in vitro* context including nanoparticle half-life, tumor vasculature leakiness and size, particle penetration through the tumor tissue, and receptor levels and availability. These variables change depending on the tumor type, nanoparticle type, the receptor being targeted, and the targeting ligand. Accordingly, it is difficult to predict how well a targeting therapy will translate from the *in vitro* to *in vivo* context. Additionally, when a targeting therapy fails to respond *in vivo* as expected, it is important to thoroughly examine the biology driving the accumulation and penetration of the therapy to determine how to design effective targeting therapeutics. For some targeting ligands, it may be necessary to examine a variety of different drug platforms or of different drug combination therapies to determine the best platform for *in vivo* work. Incorporation of targeting ligands into liposomes likely fails to improve efficacy more frequently than reported. As the field of nanomedicine progresses, it is important to examine both the successes and failures in order to understand which tumor types and patients are most likely to derive a therapeutic benefit from targeted nanoparticle therapies.

## Supporting Information

Figure S1 **Targeted H2009.1 and control liposomes accumulate in α_v_β_6_-positive H1975 tumors to the same extent.**
Subcutaneous α_v_β_6_-positive H1975 tumors were established in the right flank of NOD/SCID mice. Tumor bearing mice were injected via tail vein with either 1.3% H2009.1 tetrameric, scH2009.1 tetrameric, or naked liposomes labeled with the near infrared dye DiR. (*A*) Animals were imaged at 24, 48, and 72 hours post-liposome injection. Shown are representative images from the 72 hour time point, demonstrating that, despite the α_v_β_6_-targeting abilities of the H2009.1 peptide, all liposomes accumulate in tumors to the same extent. (*B*) At 72 hours post-liposome injection, the mice were sacrificed and the tumors and organs removed for *ex vivo* fluorescent imaging. Shown are representative images of liposome accumulation in tumors and organs. As with the *in vivo* imaging in (A), all liposomes accumulate in tumors to the same extent.
(TIF)

Figure S2 **Both targeted H2009.1 and control liposomes accumulate only in the perivasculature regions of H1975 tumors.**
Subcutaneous α_v_β_6_-positive H1975 tumors were established in the flank of NOD/SCID mice. Tumor bearing mice were injected via tail vein with either 1.3% H2009.1 tetrameric, scH2009.1 tetrameric, or naked liposomes labeled with the dye DiI. At 24 or 48 hours post-liposome injection, the mice were sacrificed and the tumors removed for sectioning and fluorescent microscopy. Blue – DAPI, Red – DiI-labeled liposomes, and Green – CD31 vasculature stain. (*A*) 10X images of liposome accumulation in tumors. The white scale bars indicate 100 μm. At both time points, all liposomes are clustered in the areas immediately adjacent to the vasculature, with the same pattern of accumulation for all of the different liposome formulations. Although there are areas of high liposome accumulation, they only occur in vascular-rich areas with large blood vessels. (*B*) Representative whole tumor image from a mouse injected with 1.3% H2009.1 tetrameric liposomes and sacrificed 24 hours after injection. The liposome accumulation overlaps with the highly vascularized periphery of the tumor. (*C*) Representative whole tumor image from a mouse injected with 1.3% naked liposomes and sacrificed 24 hours after injection. Like the targeting H2009.1 tetrameric liposomes, the naked control liposomes display the same overlap with the highly vascularized periphery of the tumor.
(TIF)

Table S1 **Quantification of accumulation of different liposome formulations in H1975 tumors as determined by whole mouse fluorescent imaging.**
(DOCX)

Table S2 **Quantification of H1975 tumor and organ accumulation of different liposome formulations as determined by *ex vivo* fluorescent imaging.**
(DOCX)
