# Supplementary figures and images for: A Liposomal Drug Platform Overrides Peptide Ligand Targeting to a Cancer Biomarker, Irrespective of Ligand Affinity or Density
Source: PLoS One. 2013 Aug 23;8(8):e72938. doi: 10.1371/journal.pone.0072938 (PMC3751880; doi:10.1371/journal.pone.0072938)

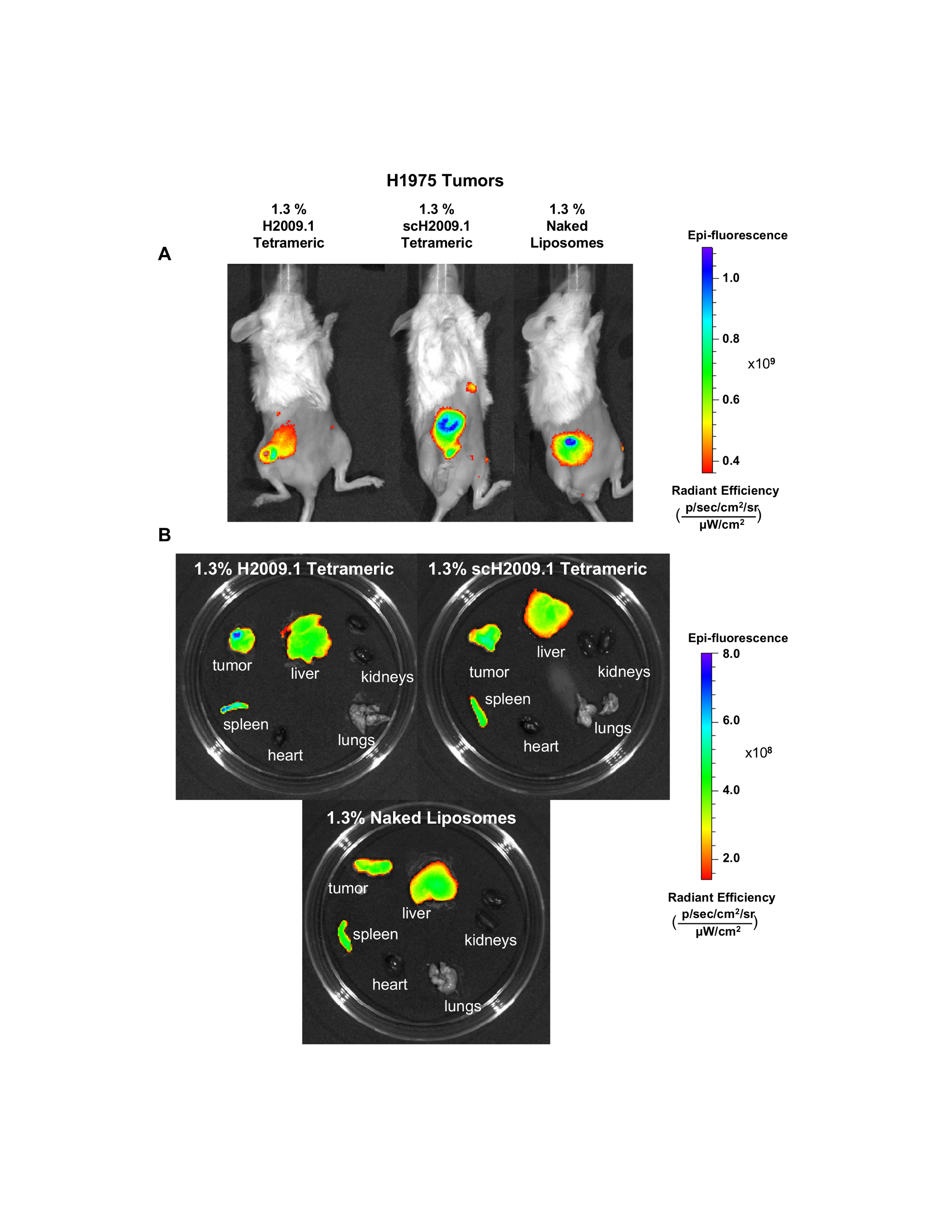

Supplement: Figure S1 — Targeted H2009.1 and control liposomes accumulate in αvβ6-positive H1975 tumors to the same extent. Subcutaneous αvβ6-positive H1975 tumors were established in the right flank of NOD/SCID mice. Tumor bearing mice were injected via tail vein with either 1.3% H2009.1 tetrameric, scH2009.1 tetrameric, or naked liposomes labeled with the near infrared dye DiR. (A) Animals were imaged at 24, 48, and 72 hours post-liposome injection. Shown are representative images from the 72 hour time point, demonstrating that, despite the αvβ6-targeting abilities of the H2009.1 peptide, all liposomes accumulate in tumors to the same extent. (B) At 72 hours post-liposome injection, the mice were sacrificed and the tumors and organs removed for ex vivo fluorescent imaging. Shown are representative images of liposome accumulation in tumors and organs. As with the in vivo imaging in (A), all liposomes accumulate in tumors to the same extent. (TIF) [file pone.0072938.s001.tif]

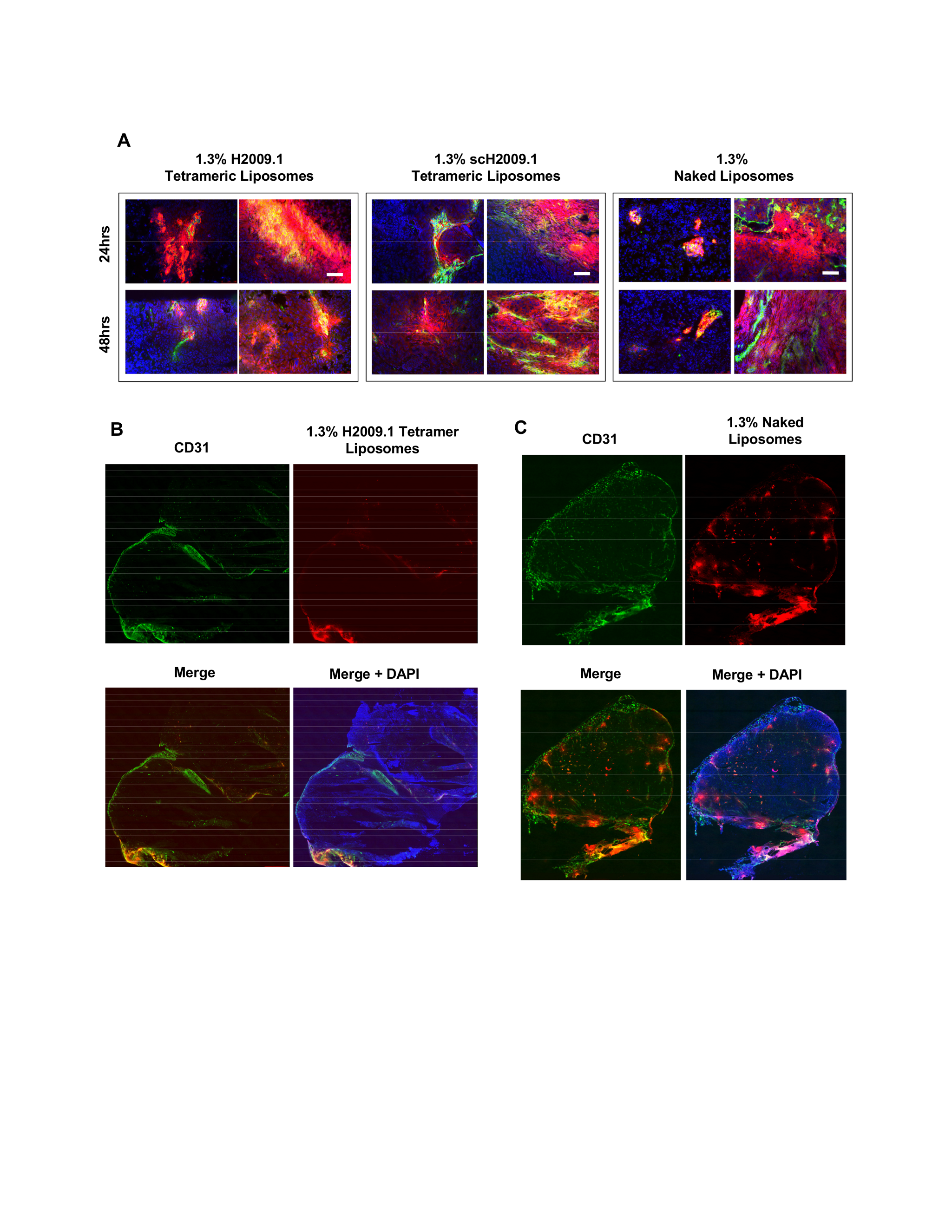

Supplement: Figure S2 — Both targeted H2009.1 and control liposomes accumulate only in the perivasculature regions of H1975 tumors. Subcutaneous αvβ6-positive H1975 tumors were established in the flank of NOD/SCID mice. Tumor bearing mice were injected via tail vein with either 1.3% H2009.1 tetrameric, scH2009.1 tetrameric, or naked liposomes labeled with the dye DiI. At 24 or 48 hours post-liposome injection, the mice were sacrificed and the tumors removed for sectioning and fluorescent microscopy. Blue – DAPI, Red – DiI-labeled liposomes, and Green – CD31 vasculature stain. (A) 10X images of liposome accumulation in tumors. The white scale bars indicate 100 μm. At both time points, all liposomes are clustered in the areas immediately adjacent to the vasculature, with the same pattern of accumulation for all of the different liposome formulations. Although there are areas of high liposome accumulation, they only occur in vascular-rich areas with large blood vessels. (B) Representative whole tumor image from a mouse injected with 1.3% H2009.1 tetrameric liposomes and sacrificed 24 hours after injection. The liposome accumulation overlaps with the highly vascularized periphery of the tumor. (C) Representative whole tumor image from a mouse injected with 1.3% naked liposomes and sacrificed 24 hours after injection. Like the targeting H2009.1 tetrameric liposomes, the naked control liposomes display the same overlap with the highly vascularized periphery of the tumor. (TIF) [file pone.0072938.s002.tif]
