# Supplementary material for: A Liposomal Drug Platform Overrides Peptide Ligand Targeting to a Cancer Biomarker, Irrespective of Ligand Affinity or Density
Source: PLoS One. 2013 Aug 23;8(8):e72938. doi: 10.1371/journal.pone.0072938 (PMC3751880; doi:10.1371/journal.pone.0072938)
Supplement: Table S1 — Quantification of accumulation of different liposome formulations in H1975 tumors as determined by whole mouse fluorescent imaging. (DOCX) [file pone.0072938.s003.docx]

Table S1. Quantification of accumulation of different liposome formulations in H1975 tumors as determined by whole mouse fluorescent imaging.

|  | **Liposome Formulation** | **Radiant Efficiency (x 10^9^)** | | |
| --- | --- | --- | --- | --- |
|  |  | **24hrs** | **48hrs** | **72hrs** |
| **H1975 tumors** | H2009.1 Tetrameric | 8.00 ± 0.810 | 9.50 ± 0.650 | 9.70 ± 1.39 |
|  | scH2009.1 Tetrameric | 10.3 ± 1.61 | 10.9 ± 2.65 | 11.1 ± 1.98 |
|  | Naked | 8.10 ± 2.32 | 9.30 ± 1.83 | 11.3 ± 1.64 |

Legend: Quantification of the tumor accumulation of DiR-labeled liposomes in α_v_β_6_-positive H1975 tumors at different time points post-liposome injection, as visualized by whole mouse fluorescent imaging in Supplementary Figure 1A.
