# Supplementary material for: A Liposomal Drug Platform Overrides Peptide Ligand Targeting to a Cancer Biomarker, Irrespective of Ligand Affinity or Density
Source: PLoS One. 2013 Aug 23;8(8):e72938. doi: 10.1371/journal.pone.0072938 (PMC3751880; doi:10.1371/journal.pone.0072938)
Supplement: Table S2 — Quantification of H1975 tumor and organ accumulation of different liposome formulations as determined by ex vivo fluorescent imaging. (DOCX) [file pone.0072938.s004.docx]

Table S2. Quantification of H1975 tumor and organ accumulation of different liposome formulations as determined by *ex vivo* fluorescent imaging.

|  | **Liposome Formulation** | **Tumor or Organ** | **Radiant Efficiency (x 10^9^)** |
| --- | --- | --- | --- |
| **H1975 tumors** | H2009.1 Tetrameric | Tumor | 3.76 ± 0.923 |
|  |  | Liver | 10.8 ± 0.305 |
|  |  | Spleen | 1.76 ± 0.195 |
|  | scH2009.1 Tetrameric | Tumor | 4.99 ± 1.57 |
|  |  | Liver | 9.06 ± 0.735 |
|  |  | Spleen | 1.88 ± 0.290 |
|  | Naked | Tumor | 3.86 ± 1.24 |
|  |  | Liver | 9.23 ± 0.428 |
|  |  | Spleen | 1.45 ± 0.170 |

Legend: Quantification of the H1975 organ and tumor accumulation of DiR-labeled liposomes after animal sacrifice at 72 hours post-liposome injection, as visualized by *ex vivo* fluorescent imaging in Supplementary Figure 1B.
